# Polyploid genome of *Camelina sativa *revealed by isolation of fatty acid synthesis genes

**DOI:** 10.1186/1471-2229-10-233

**Published:** 2010-10-27

**Authors:** Carolyn Hutcheon, Renata F Ditt, Mark Beilstein, Luca Comai, Jesara Schroeder, Elianna Goldstein, Christine K Shewmaker, Thu Nguyen, Jay De Rocher, Jack Kiser

**Affiliations:** 1 Targeted Growth, Inc., 2815 Eastlake Ave E Suite 300, Seattle, WA 98102, USA; 2 Dept. of Biochemistry/Biophysics, Texas A&M University, TAMU 2128 College Station, TX 77843, USA; 3 Plant Biology and Genome Center, 451 Health Sciences Drive, University of California Davis, Davis, CA 95616, USA; 4 BluGoose Consulting, Woodland, CA 95776, USA; 5 Sustainable Oils, LLC, 3208 Curlew St., Davis, CA 95616, USA

## Abstract

**Background:**

*Camelina sativa*, an oilseed crop in the Brassicaceae family, has inspired renewed interest due to its potential for biofuels applications. Little is understood of the nature of the *C. sativa *genome, however. A study was undertaken to characterize two genes in the fatty acid biosynthesis pathway, *fatty acid desaturase (FAD) 2 *and *fatty acid elongase (FAE) 1*, which revealed unexpected complexity in the *C. sativa *genome.

**Results:**

In *C. sativa*, Southern analysis indicates the presence of three copies of both *FAD2 *and *FAE1 *as well as *LFY*, a known single copy gene in other species. All three copies of both *CsFAD2 *and *CsFAE1 *are expressed in developing seeds, and sequence alignments show that previously described conserved sites are present, suggesting that all three copies of both genes could be functional. The regions downstream of *CsFAD2 *and upstream of *CsFAE1 *demonstrate co-linearity with the Arabidopsis genome. In addition, three expressed haplotypes were observed for six predicted single-copy genes in 454 sequencing analysis and results from flow cytometry indicate that the DNA content of *C. sativa *is approximately three-fold that of diploid *Camelina *relatives. Phylogenetic analyses further support a history of duplication and indicate that *C. sativa *and *C. microcarpa *might share a parental genome.

**Conclusions:**

There is compelling evidence for triplication of the *C. sativa *genome, including a larger chromosome number and three-fold larger measured genome size than other *Camelina *relatives, three isolated copies of *FAD2*, *FAE1*, and the *KCS17-FAE1 *intergenic region, and three expressed haplotypes observed for six predicted single-copy genes. Based on these results, we propose that *C. sativa *be considered an allohexaploid. The characterization of fatty acid synthesis pathway genes will allow for the future manipulation of oil composition of this emerging biofuel crop; however, targeted manipulations of oil composition and general development of *C. sativa *should consider and, when possible take advantage of, the implications of polyploidy.

## Background

Interest in biofuels has prompted researchers to critically evaluate alternative feedstocks for biofuel production. One important, emerging biofuel crop is *Camelina sativa *L. Cranz (Brassicaceae), commonly referred to as "false flax" or "gold-of-pleasure". Renewed interest in *C. sativa *as a biofuel feedstock is due in part to its drought tolerance and minimal requirements for supplemental nitrogen and other agricultural inputs [1,2]. Similar to other non-traditional, renewable oilseed feedstocks such as *Jatropha curcas *L. ("jatropha"), *C. sativa *grows on marginal land. Unlike jatropha, which is a tropical and subtropical shrub, *C. sativa *is native to Europe and is naturalized in North America, where it grows well in the northern United States and southern Canada.

In addition to its drought tolerance and broad distribution, several other aspects of *C. sativa *biology make it well suited for development as an oilseed crop. First, *C. sativa *is a member of the family Brassicaceae, and thus is a relative of both the genetic model organism *Arabidopsis thaliana *and the oilseed crop *Brassica napus*. The close relationship between *C. sativa *and Arabidopsis [3,4] makes the Arabidopsis genome an ideal reference point for the development of genetic and genomic tools in *C. sativa*. Second, the oil content of *C. sativa *seeds is comparable to that of *B. napus*, ranging from 30 to 40% (w/w)[5], suggesting that agronomic lessons from the cultivation of *B. napus *are applicable to *C. sativa *cultivation. Finally, the properties of *C. sativa *biodiesel are already well described [6], and both seed oil and biodiesel from *C. sativa *were used as fuel in engine trials with promising results [6,7].

Notwithstanding its potential for oil production, there is limited molecular and genomic information on this crop. Published studies detailing the biology of *C. sativa *and its closest relatives in the genus *Camelina *are few. However, several important findings can be drawn from the literature. Taxonomic treatments describe 11 species in the genus with a center of diversity in Eurasia [8], although *C. sativa*, *C. rumelica*, *C. microcarpa*, and *C. alyssum *are naturalized weeds with broad distributions. *Camelina *species can be annual or biennial, with some species requiring vernalization to induce flowering [9]. Chromosome counts range from *n *= 6 in *C. rumelica *[10,11], or *n *= 7 in *C. hispida *[12], upwards to *n *= 20 in *C. sativa*, *C. microcarpa*, and *C. alyssum *[2,13]. Some *Camelina *species are interfertile; crosses of *C. sativa *with *C. alyssum*, and *C. sativa *with *C. microcarpa*, produce viable seed [14]. In addition to these studies, a limited amount of molecular and sequence information is available for *C. sativa *[2,15-17].

Understanding the *Camelina sativa *genome is essential if agronomic properties are to be improved through molecular assisted breeding, mutation breeding, and/or genetic manipulation. For example, modification of the oil composition for superior biodiesel is a natural goal for this oilseed crop. *C. sativa *is high in polyunsaturated fatty acids such as linoleic acid (18:2; carbons:double bonds) and alpha-linolenic acid (18:3) as well as very long chain fatty acids (greater than 18 carbons) such as 11-eicosenoic acid (20:1) [18], while an ideal biodiesel blend is high in oleic acid (18:1) [19]. Target genes for modification could therefore include *FATTY ACID DESATURASE 2 (FAD2)*, a membrane bound delta-12-desaturase which converts oleic acid to linoleic acid [20-24], and *FATTY ACID ELONGASE 1 (FAE1) *which sequentially adds 2 carbon units to 18 carbon fatty acid CoA conjugates, resulting in very long chain fatty acids [25-29].

Manipulation of genes affecting traits of interest requires knowledge of their duplication status. Whole genome duplication is particularly relevant because it is common in plants, and because in the case of allopolyploidy it results in two or three independent copies of each gene. Allopolyploidy, such as found in wheat, cotton and peanut, is defined by the concurrent presence and maintenance in the same nucleus of two or more diploid genomes. In an allopolyploid, each chromosome pairs specifically to its own homolog, and not to any homoeolog, resulting in diploid inheritance [30,31]. Allopolyploids are usually formed by interspecific hybridization concurrent to genome duplication, but could also result from diploidization and divergence of genomic sets in an autopolyploid [30]. Once formed, allopolyploids are relatively stable. Gene duplicates slowly decay over millions of years back to diploidy. For example, a distinct but partial duplication pattern still detectable in the Arabidopsis genome is thought to result from an approximately 25 million year old polyploidization event [32]. The genomes of maize and soybean display widespread, but not universal duplication and are estimated to be 10 million year old polyploids [33,34]. Polyploids in which gene loss has advanced so far that duplication is no longer universal have been defined "paleopolyploids" although this term carries no precise temporal definition and could be extended to all known sequenced diploid angiosperms. Gene duplication is thus universal in a recent polypoid and becomes less and less pervasive in older polyploids as duplicates decay back to singletons. For a set of nearly 1000 genes the singleton pattern can be confirmed in all major sequenced diploid species [35].

We report the sequences of three copies of both *FAE1 *and *FAD2 *recovered from *C. sativa*. We used Southern blots to determine whether the recovered copies are allelic or if they represent multiple loci. Moreover, we performed phylogenetic analyses to infer the evolutionary history of the copies, and quantitative PCR (qPCR) to explore whether there is evidence of functional divergence among them. To better understand the *C. sativa *genome and to determine whether the multiple copies recovered are the result of polyploidization, we analyzed the genome sizes of *C. sativa *and its closest relatives in the genus *Camelina *by flow cytometry. Finally, we used next generation RNA sequencing data to demonstrate that well-characterized single-copy genes are present in triplicates. Collectively our results indicate that *C. sativa *is a hexaploid whose oil composition is likely influenced by more than one functional copy of *FAE1 *and *FAD2*. Thus in *C. sativa*, oil composition as well as other traits are likely to be determined by multiple copies of causative genes.

## Results

### Southern blot hybridizations show multiple copies of genes in *Camelina sativa*

**Figure 1:**
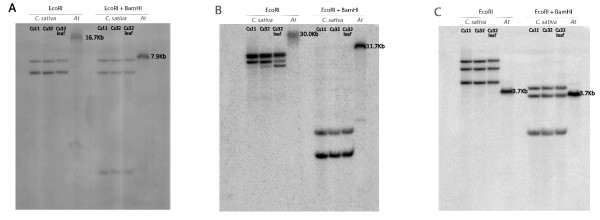
**Southern blot analysis of *Camelina sativa *and Arabidopsis**. A blot containing genomic DNA from *C. sativa *and Arabidopsis digested with EcoRI or a combination of EcoRI and BamHI was hybridized with an α-32P dCTP - labeled (A) *FAD2 *probe, (B) *FAE1 *probe or (C) *LFY *probe obtained from PCR amplification of *C. sativa *DNA. The same blot was used for all three probes. The expected sizes of the Arabidopsis fragments are indicated beside the bands and are consistent with complete digestion of the DNA.

As a first step to characterize genes involved in fatty acid biosynthesis, we determined the copy number of *FAD2 *and *FAE1 *by Southern blot analysis. Since *C. sativa *is closely related to *Arabidopsis thaliana *[3,4], we designed primers based on Arabidopsis genomic sequence that amplified conserved regions of *FAD2 *and *FAE1 *(Additional File 1). Using these primers, we PCR amplified products of 225 base pairs (bp) (*FAD2*) and 403 bp (*FAE1*) from Arabidopsis and from *C. sativa*. The *C. sativa *products were cloned, sequenced, and compared with Arabidopsis *FAD2 *and *FAE1 *sequences [36] to confirm their identities. We used the *C. sativa *fragments as probes in Southern blot experiments (Figure 1). Results of the Southern blots revealed three bands in *C. sativa *for both *FAD2 *(Figure 1A) and *FAE1 *(Figure 1B), whereas hybridization revealed only a single band in Arabidopsis for both genes (Figure 1A &1B). These results suggest that *FAD2 *and *FAE1 *occur in at least three copies in *C. sativa*, while they are single copy in Arabidopsis [36]. Fatty acid genes can be multi-copy in many species, including soybean [37], *Brassica napus *[38], olive (*Olea europaea*) [39], maize [40], and sunflower [41]. Therefore, we designed a probe for Southern blot hybridization of the gene *LEAFY *(*LFY*), which is known to be single copy in a wide variety of species from several plant families [42]. Three bands were observed following hybridization with the *LFY *probe of the same blot as was used for *FAD2 *and *FAE1*, suggesting *LFY *also exists as three copies in *C. sativa *(Figure 1C).

### Copies of *C. sativa FAD2 *and *FAE1 *are highly similar to each other and to their putative orthologs from Arabidopsis

We cloned and sequenced the full length genomic and cDNA sequences of *C. sativa FAD2 *and *FAE1*. Using primers designed from Arabidopsis *FAD2 *and *Crambe abyssinica FAE1 *(Additional File 1), we PCR amplified a band of approximately 1.2 kb for *FAD2 *and 1.5 kb for *FAE1 *from *C. sativa*. For each gene, we sequenced more than 60 clones. Three different versions of both *CsFAD2 *and *CsFAE1 *were recovered and designated A, B, and C. It should be noted that the A, B, and C copies were named independently for *CsFAD2 *and *CsFAE1*, and thus are not associated with a particular genome.

The three copies of *C. sativa FAD2 *are 1155 bp long, lack introns in the coding regions, are 97% identical at the nucleotide level, and encode proteins that are 99% identical in sequence (Table 1). One of the *CsFAD2 *copies, *CsFAD2 *A, contains a BamHI site (see Additional File 2), and thus this copy likely produced the smallest fragment in the Southern blot hybridization of *FAD2 *(Figure 1A; BamHI + EcoRI digest). The *C. sativa *nucleotide sequences of *FAD2 *are greater than 93% identical to Arabidopsis *FAD2*, and the putative encoded proteins from the two species share greater than 96% identity (Table 1).

**Table 1:** Nucleotide and amino acid identity of *Camelina sativa *and *Arabidopsis thaliana *FAD2 and FAE1 genes

Gene		% Nucleotide Identity *				% Amino Acid Identity			
		**AtFAD2**	**CsFAD2 A**	**CsFAD2 B**	**CsFAD2 C**	**AtFAD2**	**CsFAD2 A**	**CsFAD2 B**	**CsFAD2 C**

**FAD2**	**AtFAD2**	100	93.6	93.8	93.4	100	96.9	96.6	96.4

	**CsFAD2 A**		100	97.3	98.3		100	99.0	99.5

	**CsFAD2 B**			100	97.7			100	99.5

	**CsFAD2 C**				100				100

		**AtFAE1**	**CsFAE1 A**	**CsFAE1 B**	**CsFAE1 C**	**AtFAE1**	**CsFAE1 A**	**CsFAE1 B**	**CsFAE1 C**

**FAE1**	**AtFAE1**	100	90.7	91.2	91.0	100	91.9	91.7	91.7

	**CsFAE1 A**		100	97.8	96.8		100	97.6	96.4

	**CsFAE1 B**			100	97.2			100	96.8

	**CsFAE1 C**				100				100

*Nucleotide identity is in coding region only.

The 5' untranslated region (utr) was recovered for all three copies of *CsFAD2 *by rapid amplification of cDNA ends (RACE) PCR. We then used primers designed from the 5' utr sequence (Additional File 1) to amplify an approximately 1.4 kb intron found within the 5' utr from all three copies of *C. sativa FAD2*. A similarly sized intron is present in Arabidopsis [36] and in *Sesamum indicum *(sesame) where it has been shown to be involved in regulating *FAD2 *expression [43].

All three copies of *FAE1 *in *C. sativa *are 1518 bp long and lack introns. When the nucleotide sequences and the putative encoded proteins of the three copies are compared they are more than 96% identical (Table 1). In comparison to Arabidopsis, the nucleotide sequences are more than 90% identical, while the encoded proteins are more than 91% identical (Table 1). Thus, the three copies of *C. sativa FAD2 *and the three copies of *FAE1 *are highly similar to each other and to their putative orthologs from Arabidopsis.

### Alignments of FAD2 and FAE1 protein sequences from several species reveal conserved and non-conserved domains

**Figure 2:**
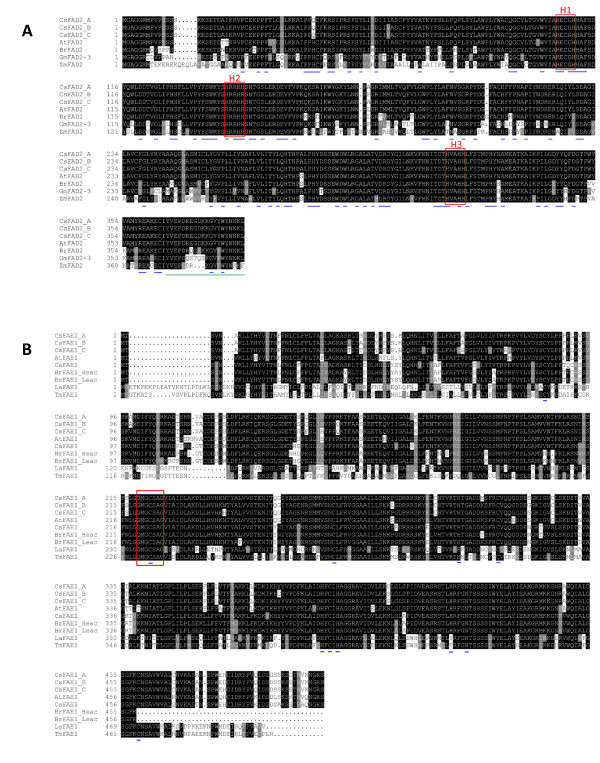
**FAD2 and FAE1 protein alignment**. (A) Amino acid sequence comparison of the three *Camelina sativa *FAD2 sequences, *Arabidopsis thaliana *FAD2 sequence [Genbank: NP_187819], *Brassica rapa *FAD2 sequence [Genbank: AJ459107], *Glycine max *FAD2-3 sequence [Genbank: DQ532371], *Zea mays *FAD2 sequence [Genbank: AB257309]. Blue underlines below the sequences indicate amino acids conserved in all 50 FAD2 sequences compared by Belo *et al*.[46] while the green underline indicates the ER localization signal [45]. The three His boxes described by Tocher *et al *[44] are indicated with red boxes. (B) Amino acid sequence comparison of the three *Camelina sativa *FAE1 sequences, *Arabidopsis thaliana *FAE1 [Genbank:NP_195178], *Crambe abyssinica *[Genbank: AAX22298], *Brassica rapa *Heac FAE1 [Genbank: Y14975], *Brassica rapa *Leac FAE1 [Genbank: Y14974], *Limnanthes alba *(meadow foam) [Genbank: AF247134] and *Tropaeolum majus *(nasturtium) [Genbank: ABD77097]. Blue underlines below the sequence indicate the asparagine at position 424 and the highly conserved histidine and cysteine residues described by Ghanevati and Jaworski [49,50]. The red box indicates the region highly conserved among condensing enzymes in very long chain fatty acid biosynthesis [62] Abbreviations: Heac = High erucic acid, Leac = Low erucic acid.

We aligned translated amino acid sequences from the three copies of *C. sativa *FAD2 with the FAD2 protein sequences from Arabidopsis; *Brassica rapa*, an agronomically important member of the Brassicaceae family; *Glycine max*, an agronomically important dicot; and *Zea mays*, an agronomically important monocot (Figure 2A). All three copies of *C. sativa *FAD2 have the three conserved HIS boxes found in all membrane-bound desaturases [44] as well as the ER localization signal described by McCartney *et al *[45]. Furthermore, the conserved amino acids identified in an alignment of the FAD2 sequences from 34 different species [46] are also present in *C. sativa *with the exception of a positively-charged histidine at position number 44, which is substituted by a polar, uncharged glutamine in *C. sativa*. When we amplified the *FAD2 *gene from several *Camelina *and outgroup species and aligned the translated amino acid sequences, we found that the FAD2 proteins from *Capsella rubella*, *Camelina microcarpa*, *Camelina laxa*, and one copy from *Camelina rumelica *contain a glutamine at amino acid position 44, while the FAD2 proteins from *Arabidopsis lyrata*, *Camelina hispida*, and a second copy from *Camelina rumelica *contained a histidine (Additional File 3).

We aligned the translated amino acid sequences from the three copies of *C. sativa *FAE1 with the seed-specific FAE1 proteins from Arabidopsis, *Crambe abyssinica*, a high and low erucic acid *Brassica rapa*, *Limnanthes alba*, and *Tropaeolum majus *(Figure 2B). *L. alba *and *T. majus *are both in the order Brassicales and their seeds accumulate high levels of very long chain fatty acids [47,48]. Four conserved histidine residues and six conserved cysteine residues, including the active site at cysteine 223, as well as an asparagine residue at 424 required for FAE1 acitivity were previously identified by Ghanevati and Jaworski [49,50]. All conserved residues were found to be present in all three copies of *C. sativa *FAE1. More differences were apparent between the three *C. sativa *FAE1 sequences and the other FAE1 sequences than observed in the FAD2 comparison (Figure 2A and 2B), an observation consistent with the level of amino acid identity seen between Arabidopsis and *C. sativa *FAD2 versus FAE1 (Table 1).

### All three copies of *FAD2 *and *FAE1 *are expressed in developing seeds of *C. sativa*

**Figure 3:**
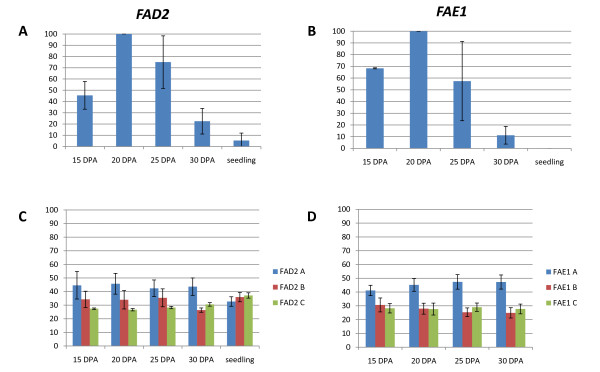
***FAD2 *and *FAE1 *expression in developing seeds**. Relative combined expression of all three copies of (A) *CsFAD2 *and (B) *CsFAE1 *measured by real time qPCR at 15, 20, 25, and 30 days post anthesis (DPA) and in 2 week old seedlings. The 20 DPA sample, which expressed *CsFAD2 *and *CsFAE1 *at the highest amount, was arbitrarily set to 100% and used as the calibrator for the remaining samples. Error bars represent the standard deviation of 3 replicate experiments. Sequenom SNP analysis demonstrating the expression of each version of (C) *CsFAD2 *or (D) *CsFAE1 *relative to the other versions. Error bars represent the standard deviation of three (for *CsFAD2*) or four (for *CsFAE1*) SNP analyses. Because *FAE1 *is not expressed in *C. sativa *seedlings (B), the relative expression of the 3 copies of *CsFAE1 *in seedling tissue is not shown (D).

The conservation of amino acids as well as the presence of the 5' regulatory intron in *CsFAD2 *suggests that all three copies of *CsFAD2 *and *CsFAE1 *could be functional. To determine whether these genes are also expressed, we first evaluated total *CsFAD2 *and *CsFAE1 *gene expression in developing seeds and in seedling tissue using real time quantitative PCR (qPCR) with primer/probe combinations designed to detect all three copies of each gene (Additional File 4). *CsFAD2 *expression in seedling tissue is present but minimal (0.4% of that seen in seeds at 20 days post-anthesis (DPA)), while *CsFAE1 *expression could not be detected in seedlings (Figure 3A and 3B). In developing seeds, both *CsFAD2 *and *CsFAE1 *expression peaks at 20 DPA and is reduced by 30 DPA (Figure 3A and 3B). In Arabidopsis, *FAD2 *peaks earlier and decreases sooner than *FAE1 *[51].

We wondered whether the expression of each of the *FAD2 *and *FAE1 *copies present in *C. sativa *are equally or differentially expressed in the seed. Duplicated genes are frequently silenced either throughout the plant or in a tissue-specific manner [52-55]; hence we hypothesized that one or more of the copies of each gene could be significantly down-regulated. We used the Sequenom MassARRAY™ method for determining allele-specific expression of a gene [56] to evaluate the relative expression of each of the copies of *CsFAD2 *and *CsFAE1*. We identified at least three single nucleotide polymorphisms (SNPs) specific to each of the *CsFAD2 *A, B, and C and the *CsFAE1 *A, B, and C copies (Additional File 5) and then calculated the frequency of each SNP in seed cDNA. Controls consisting of the cloned *CsFAE1 *A, B, and C copies combined to known frequencies showed that the method is greater than 80% accurate (data not shown). No evidence of silencing of any particular copy of either *CsFAE1 *or *CsFAD2 *was discovered. We did observe differential expression, especially of *CsFAE1 *A, which accounts for approximately 40-50% of *CsFAE1 *expression in seeds at 20-30 DPA (Figure 3C and 3D).

### Characterization of sequences upstream of *C. sativa FAE1 *and downstream of *C. sativa FAD2 *suggests colinearity with Arabidopsis

**Figure 4:**
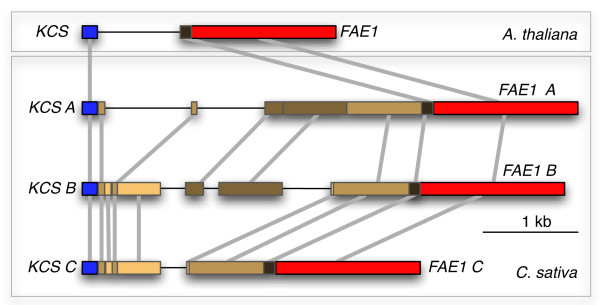
**Structure and conservation of the *KCS17-FAE1 *intergenic region in *Camelina sativa***. The three putative homoeologous regions in *C. sativa *are aligned to the orthologous region of Arabidopsis to display blocks of homology identified on a dot matrix by perfect conservation of a sliding window of 9 bases. The *KCS17 *and *FAE1 *gene, respectively blue and red, flank a variable region in which potentially related sequences are marked by different shades of brown, corresponding to varying levels of similarity, which were estimated visually from dot matrix plots since proper alignment was not possible. Lined regions display reduced or no conservation. The large variation in the intergenic region of the triplicated *KCS17-FAE1 *DNA of *C. sativa *is consistent with independent evolution before reunion of diverged genomes by polyploidization.

To investigate whether the different copies of *C. sativa FAD2 *and *FAE1 *are the result of allelic variation or are in fact independent loci, we obtained sequence from the region upstream of *CsFAE1 *and downstream of *CsFAD2*. Assuming colinearity between *C. sativa *and Arabidopsis for the region around *FAE1*, we PCR amplified the region 5' to *CsFAE1 *using a forward primer for the upstream gene *KCS17 *with reverse primers for *C. sativa FAE1 *(Additional File 1). The resulting sequences we obtained for the putative *C. sativa KCS17 *were highly similar to the last 189 bp of Arabidopsis *KCS17*, suggesting that we had in fact amplified the orthologous *C. sativa *region upstream of *FAE1*, confirming colinearity between the two species. We then used a dot plot [57] to compare the three *C. sativa *upstream sequences to each other and to Arabidopsis with parameters set for perfect match on a sliding window of 9 bases (Additional File 6). The coordinates from the dot plot were used to define blocks of homology between Arabidopsis and the three *C. sativa *copies (Figure 4). The results show a variable intergenic region containing potentially related blocks common to two or more genomes.

Colinearity with Arabidopsis was also found for a region downstream of *FAD2 *containing the *ACTIN11 *(*ACT11*) gene for two out of the three *C. sativa *copies (data not shown). For the third copy, the region downstream of *CsFAD2 *A could have been missed if the length of the amplified product was too large. Alternatively, the region downstream of *CsFAD2 *A might not exhibit colinearity with Arabidopsis and the possibility remains that two of the copies of *CsFAD2 *result from a tandem gene duplication.

### Deep sequencing of *Camelina sativa *developing seed transcriptome reveals three expressed haplotypes for predicted single-copy genes

**Figure 5:**
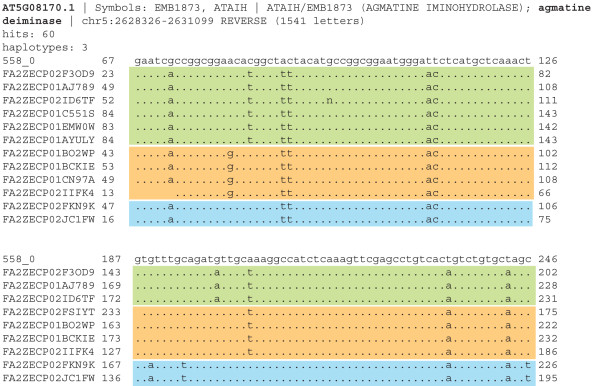
**Deep sequencing reads for *C. sativa *agmitine deiminase**. Sequences identified from 454 sequencing of cDNA from 15 DPA *C. sativa *seeds, aligned with the Arabidopsis agmatine deiminase cDNA (At5G08170). The three haplotypes are evident and indicate that three different copies of this "single-copy gene" are present and expressed in the *C. sativa *genome, presumably representing three homoeologs. Similar results were obtained with the other 5 single-copy genes sampled (Additional File 7). The degree of nucleotide similarity estimated in these aligned regions (3-6%) is consistent with the diversity observed in *FAD2 *and *FAE1 *loci.

To further explore the *C. sativa *genome, we determined the haplotype number of predicted single-copy genes in a 454 sequencing data set of cDNA expressed in 15 DPA *C. sativa *seeds. The reads were aligned to 956 genes identified by Duarte *et al*. [35] as single-copy genes shared in flowering plants. The six genes with the highest coverage (> 60 reads per gene) were selected for further evaluation. Remarkably, all 6 genes examined showed expression of three clear haplotypes (Additional File 7) as exemplified by the agmatine deiminase gene (Figure 5), indicating that the triplication of the genes in the *C. sativa *genome is common and not limited to *FAD2*, *FAE1*, and *LFY*. When the genomic status of the same 6 genes was examined in the genomes of paleopolyploids such as maize and soybean, whose genome duplication is about 10 million years old [33,34], only a subset of these genes was retained as duplicates (Table 2). This lack of duplication in maize and soybean contrasted with the consistent pattern of triplication in *C. sativa*.

**Table 2:** Number of observed haplotypes for predicted single-copy genes in *Arabidopsis thaliana, Camelina sativa, Zea mays*, and *Glycine max*

	# haplotypes			
	
	***Arabidopsis thaliana***^***1***^	***Camelina sativa***^***2***^	***Zea mays***^***3***^	***Glycine max***^***4***^
**At1G31600**	1	3	1	1

**At1G65270**	1	3	1	2

**At2G18040**	1	3	2	2

**At4G37830**	1	3	1	2

**At5G08060**	1	3	2	2

**At5G08170**	1	3	1	1

^1 ^Arabidopsis genome sequencing project [http://www.arabidopsis.org]

^2^Expressed cDNA, this study

^3^maize genome sequencing project [http://www.maizesequence.org]

^4^soybean genome sequencing project [http://www.phytozome.org]

### The genomes of *C. sativa*, *C. alyssum*, and *C. microcarpa *are larger than the genomes of other *Camelina *species

**Figure 6:**
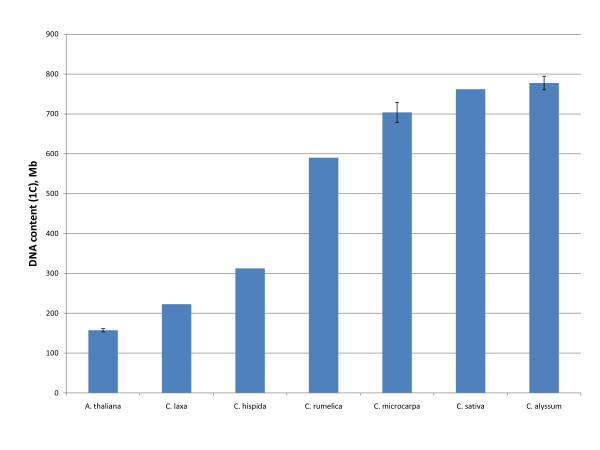
**Genome content of *Camelina *species**. 1C nuclei were stained with propidium iodide and analyzed by flow cytometry. Error bars represent the standard deviation of 2-4 replicate samples.

We calculated DNA content in several accessions of *C. sativa *and related species from flow cytometry analyses using propidium iodide-stained nuclei. We used Arabidopsis accession Col-0 (2X) and its tetraploid (4X) derivative as genome size standards. *C. sativa*, *C. alyssum*, and *C. microcarpa *diploid (2C) genomes had a haploid content between 650 and 800 Mb (Figure 6). *C. sativa *accessions uniformly displayed a genome size close to 750 Mb. North American isolates of *C. sativa*, *C. alyssum*, and *C. microcarpa *have reported chromosome counts of *n *= 20 [13]. The genomes of *C. rumelica *(600 Mb), *C. hispida *(300 Mb) and *C. laxa *(210 Mb) are smaller than those of *C. sativa*, *C. alyssum*, and *C. microcarpa*. Chromosome counts of both *n *= 6 [10,11] and *n *= 12 [12] have been recorded for *C. rumelica*, while only a single count of *n *= 7 exists for *C. hispida *[12]. To our knowledge, no published counts exist for *C. laxa*.

### Phylogenetic analysis of *FAD2 *and *FAE1 *indicate that *C. sativa *and *C. microcarpa *are closely related

**Figure 7:**
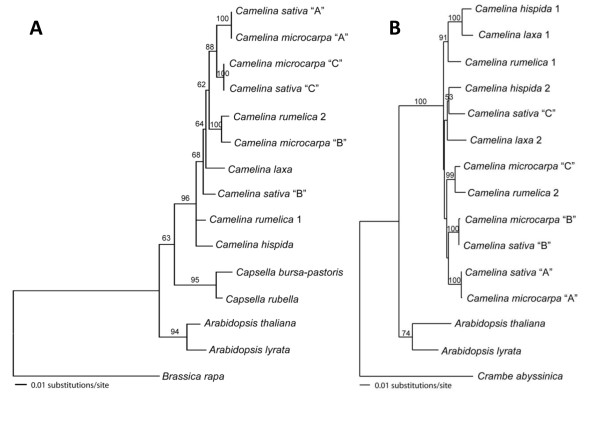
**Phylogenetic analyses of Camelineae *FAD2 *and *FAE1***. Maximum-likelihood trees showing branch length and bootstrap support (100 bootstrap replicates) for (A) 15 *FAD2 *sequences from five species of *Camelina *and five outgroup species calculated using the TVM+I+Γ model in PAUP* and rooted with *Brassica rapa FAD2 *(-LnL 3665.277); and for (B) 15 *FAE1 *sequences from five species of *Camelina *and three outgroup species calculated using the HKY+I+Γ model in PAUP* and rooted with *Crambe abyssinica FAE1 *(-LnL 5051.552). Sequences obtained from Genbank are *Capsella bursa-pastoris FAD2 *[Genbank: DQ518293], *Arabidopsis thaliana FAD2 *[Genbank: NM_112047], *Brassica rapa FAD2 *[Genbank: AJ459107], *Arabidopsis thaliana FAE1 *[Genbank: NM_119617], and *Crambe abyssinica FAE1 *[Genbank: AY793549].

To understand the duplication history of the multiple *FAD2 *and *FAE1 *copies recovered from *C. sativa*, we amplified the *FAD2 *and *FAE1 *genes from several *Camelina *species and outgroup species, and inferred phylogeny for each gene. The sampling of taxa chosen allowed us to test whether *FAD2 *and *FAE1 *duplication events occurred after *Camelina *diverged from its closest relatives or within the genus. Results from the evaluation of 55 different models of sequence evolution using Modeltest 3.7 [58] indicated that the *FAD2 *sequence data are best described by the TVM+I+Γ model, while the *FAE1 *data are best described by the HKY+I+Γ model. Likelihood phylogenetic analyses in PAUP* 4.b [59] produced a single *FAD2 *tree (-LnL 3665.277; Figure 7A), and a single *FAE1 *tree (-LnL 5051.552; Figure 7B).

Phylogenies inferred from *FAD2 *and *FAE1 *data indicate a history of duplication for both markers. Both *C. microcarpa *and *C. sativa *have three distinct copies of *FAD2 *and *FAE1*. Moreover, for *FAD2*, the A and C copies from these two species are monophyletic with strong (100%) bootstrap support (bs); for *FAE1 *the A and B copies from these species are strongly monophyletic (100% bs). In contrast, neither the *FAD2 *B copies of *C. sativa *and *C. microcarpa*, nor the *FAE1 *C copies of these species form a monophyletic group with each other. Instead, our results indicate that *C. rumelica *has two distinct copies of *FAD2 *and that one of these copies (*FAD2*-2) is strongly monophyletic with *C. microcarpa FAD2 *B. We recovered only a single *FAD2 *copy for *C. laxa *and *C. hispida*. In contrast, we recovered at least two distinct copies of *FAE1 *from all sampled *Camelina *species. The *FAE1*-1 copy of *C. laxa*, *C. hispida*, and *C. rumelica *form a monophyletic group (91% bs), with the former two species sister to one another with strong support (100% bs). Similar to the results from *FAD2*, *C. rumelica FAE1*-2 is sister to one of the *C. microcarpa *copies (*FAE1 *C; 99% bs). Neither the *C. sativa FAD2 *B copy, nor the *C. sativa FAE1 *C copy, shows a well supported sister relationship to other *FAD2 *or *FAE1 *sequences. However, in the *FAE1 *tree, *C. sativa FAE1 *C is very weakly supported as sister to *C. hispida FAE1*-2 (53%). Finally, all recovered *FAD2 *and *FAE1 *copies from species of the genus *Camelina *are monophyletic and sister to other sampled members of the tribe Camelineae, consistent with phylogenies based on other markers [3,4].

## Discussion

*Camelina sativa *is a re-emerging oilseed with tremendous potential as an alternative biofuel crop and for which genomic information is becoming increasingly available. We have obtained molecular data for nine genes, characterized in detail two genes encoding fatty acid biosynthesis enzymes and, in the process, have discovered unexpected complexity in the *C. sativa *genome.

The close relationship between *C. sativa *and the model plant *Arabidopsis thaliana *[3,4] facilitates the manipulation of known pathways, such as the one regulating fatty acid biosynthesis. *C. sativa *seed oil is high in both polyunsaturated and long chain fatty acids [5,60,61], suggesting that both CsFAD2 and CsFAE1 are present and active. Three copies each of the *FAD2 *and *FAE1 *genes were isolated from an agronomic accession of *C. sativa *using primers designed from *A. thaliana *or *Crambe abyssinica *sequence. Previously identified conserved sites in CsFAD2 [44-46] and CsFAE1 [49,50,62] are present in all three copies of each gene and a 5' intron shown to be important in regulating *FAD2 *expression in sesame [43] was identified in all three *CsFAD2 *copies. Real time qPCR data and Sequenom MassARRAY SNP analysis of the *CsFAD2 *and *CsFAE1 *cDNA showed that all three copies of each gene are expressed in developing seeds. Thus, it seems likely that all three copies of FAD2 and FAE1 in *C. sativa *are functional.

The cloning of three copies of *FAD2 *and *FAE1 *from the *C. sativa *genome, as well as the observation of three LFY hybridization signals by Southern analysis and three expressed haplotypes for 6 more predicted single-copy genes in developing seeds, could be explained by at least two possible scenarios: segmental duplications of selected regions within a diploid genome either through tandem duplications or through transpositions, or whole genome duplications resulting from polyploidization. Segmental duplications or transpositions affecting all nine examined loci are improbable compared with the explanation of polyploidy. Furthermore, no evidence of recent segmental duplication involving multiple genes has been observed in sequenced plant genomes [36,63-65].

Triplication of the *C. sativa *genome therefore likely occurred through whole genome duplication, either through autopolyploidization or through allopolyploidization. An autopolyploidy event might have triplicated a single diploid genome resulting in an autohexaploid with a haploid genome of 18, 21, or 24 chromosomes. Given that *C. sativa *has a chromosome count of *n *= 20, chromosome splitting or fusion could then have increased the chromosomes from 18 to 20, or decreased the chromosomes from 21 or 24 to 20.

Alternatively, triplication of the *C. sativa *genome might have resulted from two allopolyploidy events, resulting in first a tetraploid then a hexaploid, similar to the origin of cultivated wheat. According to this hypothesis, the three copies of each gene diverged in different diploid genomes before converging through polyploidy events. Taking into consideration the reported chromosome counts of various *Camelina *species, the basal chromosome number of the diploid parental species contributing to the *C. sativa *haploid genome of 20 chromosomes could be 7+7+6 or 8+6+6. The allopolyploid hypothesis is supported by the observation that *C. sativa *demonstrates diploid inheritance [2,66], as would be expected for an allopolyploid [31]. A hexaploid *C. sativa *could also be derived from the combination of an autotetraploid and a diploid species if, in an autopolyploidized genome, homologous chromosomes differentiated so that the subsequent chromosome-specific pairing mimicked an allopolyploid genome in its diploid inheritance patterns. Regardless of its evolutionary path, the *C. sativa *genome appears organized in three redundant and differentiated copies and can be formally considered to be an allohexaploid.

Results from our phylogenetic analyses support a history of duplication for both *FAD2 *and *FAE1 *in *Camelina*. For *FAD2*, duplications were only recovered for *C. sativa*, *C. microcarpa*, and *C. rumelica*. These data are consistent with genome size data, which indicate that all three genomes are larger than *C. laxa *and *C. hispida*, from which only a single *FAD2 *copy was recovered. Taken together, the results suggest that *C. sativa*, *C. microcarpa*, and *C. rumelica *are likely polyploids. Given the slightly smaller genome size of *C. rumelica*, and the fact that we recovered only two *FAD2 *copies from it, the *C. rumelica *sampled may be tetraploid while *C. sativa *and *C. microcarpa *are hexaploid. Interestingly, in both the *FAD2 *and *FAE1 *trees, one copy each of *C. rumelica *and *C. microcarpa *are strongly supported as sister. Thus, trees from these genes indicate that *C. rumelica *and *C. microcarpa *are closely related. The various placement of *C. microcarpa FAD2 *and *FAE1 *copies can be explained if *C. microcarpa *is the result of a hybridization event between *C. rumelica *and a currently unsampled, and thus unidentified species of *Camelina*. Two of the three copies of both *FAD2 *and *FAE1 *are identical, or nearly identical, in *C. sativa *and *C. microcarpa*, suggesting that *C. sativa *and *C. microcarpa *share a parental genome. Thus, we suggest that a *Camelina *species we did not sample contributed its genome to the hybrid formation of both *C. sativa *and *C. microcarpa*. In the case of *C. microcarpa*, the hybridization event likely involved *C. rumelica*. Given the chromosome count of *n *= 6 for *C. rumelica*, we expect the other putative parent to have an *x *= 7 genome, and furthermore to be tetraploid at *n *= 14. Such a cross would result in the observed *C. microcarpa *genome, with chromosome count *n *= 20. Interestingly, *C. hispida *is the only species we sampled with a chromosome count of *n *= 7, however no strong relationship between *C. hispida *and *C. microcarpa *is inferred in either gene tree. However, we do infer a weak relationship between *C. sativa *and *C. hispida *in the *FAE1 *tree, and thus the possibility that *C. hispida *is involved in the polyploid formation of *C. sativa *should be explored further.

What is the age of the polyploidization events likely to have formed the *C. sativa *genome? A complete answer will require a better understanding of its genome, but two findings suggest a recent origin. First, the chromosome number of *C. sativa *is inconsistent with extensive karyotype evolution and likely represents the sum of the ancestral contributions. Second, paleopolyploids such as soybean and maize display duplication of many, but not all genes as a sizeable number have decayed to singleton state. In contrast, the presence of triplicates for nine test genes of *C. sativa *is consistent with high retention of duplicates, as expected in recent polyploids.

The likely allohexaploid nature of the *Camelina sativa *genome has multiple implications. Its vigor and adaptability to marginal growth conditions may result at least in part from polyploidy. Polyploids are thought to be more adaptable to new or harsh environments, with the ability to expand into broader niches than either progenitor [67,68]. Indeed, *C. hispida *and *C. laxa*, both of which are likely diploids, are found only in Turkey, Iran, Armenia, and Azerbaijan, while *C. microcarpa *and *C. sativa *are distributed throughout Asia, Europe, and North Africa and are naturalized in North America [8,69]. The mechanisms behind this increased adaptability are not completely understood, but have been attributed to heterosis, genetic and regulatory network redundancies, and epigenetic factors [30,70].

Allohexaploidy might also affect any potential manipulations of the *C. sativa *genome, such as introgression of germplasm or induced mutations. Introgression of an exotic germplasm could be facilitated by the type of polyploidy-dependent manipulations that are possible in wheat, a potentially comparable allohexaploid [71,72]. In addition, polyploids have displayed excellent response to reverse genomics approaches such as Targeting Induced Local Lesions inGenomes (TILLING) [73,74]. As in wheat, any recessive induced mutations could be masked by redundant homoeologous loci that have maintained function [75,76]. This mutation masking implies that multiple knockout alleles at different homoeologous sites can be combined to achieve partial or complete suppression of a targeted function [77,78]. We also expect that single locus traits, whether transgenic or not, will display diploid inheritance due to preferential intragenomic pairing.

In a hexaploid oilseed crop such as *C. sativa*, manipulations of oil composition and/or yield should therefore be possible through transgenic or reverse genetic approaches, or through other genome manipulations similar to those performed in wheat. For example, the characterization of *FAD2 *and *FAE1 *in *C. sativa *could enable the use of TILLING techniques to isolate *C. sativa *plants with mutations in each of the three identified copies of both genes. We expect these mutations to result in plants with reduced levels of polyunsaturated fatty acids or long chain fatty acids, possibly in a dosage dependent manner. This will allow us to manipulate the seed oil composition of *C. sativa*, potentially creating a broad spectrum of *C. sativa *varieties possessing useful biodiesel properties, thereby further increasing the utility of this emerging biofuel crop.

## Conclusions

The discovery of triplication and divergence of genes that in known diploids are present in single copy, the cytometrically determined genome size of *Camelina *species, the pattern of relationship and inferred duplication history in the gene trees, together with the previously known chromosome counts for this taxon, indicate a likely allohexaploid genomic constitution. The characterization of genes encoding key functions of fatty acid biosynthesis lays the foundation for future manipulations of this pathway in *Camelina sativa*. Targeted manipulations of oil composition and general development of this crop, however, need to consider the implications of polyploidy and when possible take advantage of this common condition in crop plants.

## Methods

### Southern blot

*Camelina sativa *Cs11 and Cs32, and *Arabidopsis thaliana *ecotype Col-0 (Additional File 8) seeds were germinated on Arabidopsis Growth Media (1× Murashige and Skoog (MS) mineral salts, 0.5 g/L MES, 0.8% PhytaBlend™ all from Caisson Labs, North Logan, UT; pH5.7) and allowed to grow for ~2 weeks under 16/8 hours day/night, 22/18°C and ~130 μE m^-2 ^s^-1 ^light intensity. A third *Camelina sativa *sample consisted of Cs32 leaf tissue from a fully grown plant (~1 month old) that allowed us to obtain a larger amount of DNA from a single plant. Genomic DNA was isolated according to the CTAB method [79] and 10 μg was digested overnight (~16 h) with EcoRI or a combination of EcoRI plus BamHI. DNA electrophoresis and blotting were carried out using standard molecular biology techniques [80]. The probe was labelled with α-32P dCTP according to instructions of the DECAprime II kit (Ambion, Austin, TX). Hybridization was carried out overnight at 42°C. The blot was washed (30 minutes each) at 42°C in 2 × SSC, 0.1% SDS, followed by 55°C in 2 × SSC, 0.1% SDS, and then 55°C in 0.1 × SSC, 1% SDS, and exposed to a phosphorimager screen. The same blot was hybridized with different probes after stripping the membrane in boiling 0.1% SDS for 20 minutes each time.

### Cloning of *C. sativa FAD2 *and *FAE1 *genes and upstream regions

*FAD2 *and *FAE1 *genes were amplified from *C. sativa *Cs32 DNA isolated as described above, using Pfu DNA polymerase (Stratagene, La Jolla, CA) and the primers listed in Additional File 1 with a PCR machine set for 30 cycles at 58°C annealing temperature and extension time of 3 minutes. For *FAD2*, buffer A from the SureBand PCR optimization kit (Bioline, Tauton, MA) was used. All intergenic regions were isolated using Phusion polymerase (New England Biolabs, Ipswich, MA). For the initial clones of the *CsKCS17*-*CsFAE1 *intergenic region, as well as the *CsFAD2*-*CsACT11 *intergenic region, the Phusion polymerase 3-step PCR protocol with an annealing temperature of 60°C, an extension time of 3 minutes, and 40 cycles was used. A Phusion polymerase 3-step PCR with annealing temperature of 60°C, extension time of 1 minute, and 30 cycles was used to obtain more clones for *CsKCS17-CsFAE1 *intergenic regions "B" and "C", while an annealing temperature of 55°C, extension time of 2 minutes and 30 cycles was used to obtain *CsKCS17-CsFAE1 *intergenic region "A". RACE PCR was performed using the SMART™ RACE cDNA Amplification kit and Advantage 2 Polymerase (Clontech, Mountain View, CA) according to the accompanying directions. All the amplified fragments were cloned using the Zero Blunt PCR Cloning kit (Invitrogen, Carlsbad, CA.)

### FAD2 and FAE1 sequence alignments

Translated amino acid FAD2 and FAE1 sequences were aligned with AlignX (Invitrogen), with a gap opening penalty of 15, a gap extension penalty of 6.66, and a gap separation penalty range of 8. Alignments were imported into Boxshade [81] to highlight the conserved residues.

### RNA isolation and cDNA preparation

*C. sativa *Cs32 plants were grown under 24/18°C day/night conditions with a 16/8 hour photoperiod. Flowers were tagged and embryos harvested at the time points indicated. RNA was then isolated using the urea LiCl method described by Tai *et al *[82]. cDNA were prepared from 0.5 μg of DNAsed RNA that was reverse transcribed with the High Capacity cDNA RT kit (Applied Biosystems, Foster City, CA) using random primers according to the manufacturer's instructions.

### Real time quantitative PCR (qPCR)

Relative expression of *CsFAD2 *and *CsFAE1 *cDNA was measured by real time qPCR and calculated according to the comparative C_T _method (2^-ΔΔCT^). In brief, separate reactions were prepared in duplicate or triplicate for each of the genes to be measured. Each reaction contained 8 μl of the appropriate primers (200 nM each) and probe (900 nM) listed in Additional File 4 for *CsACTIN *(reference gene) or *CsFAD2 *or *CsFAE1 *(target genes); 10 μl of Applied Biosystems 2× fast Taqman PCR mix; 2 μl of cDNA. The reactions were run on an Applied Biosystems 7900HT according to the manufacturer's fast PCR method.

### Relative expression analysis

Three single nucleotide polymorphisms (SNPs) for each of *CsFAD2 *A, B, and C and *CsFAE1 *A, B, and C were identified. Each identified SNP distinguishes one copy from the other two. An additional SNP, which distinguishes *FAE*1 A, B, and C copies from each other, was also identified (Additional File 5). SNP frequencies were determined in cDNA isolated as described above by the Sequenom MassARRAY™ allele-specific expression analysis method with no competitor, as described in Park *et al *[56].

### 454 pyrosequencing

Approximately 150 μg of total RNA from 15 DPA *Camelina sativa *CS32 seed was isolated as described above and sent to Agencourt Bioscience (now known as Beckman Coulter Genomics, Danvers, MA) for isolation of mRNA, library construction and 454 sequencing, according to their established protocols.

### Analysis of "single-copy" genes

The cDNA sequences of the 956 single copy genes were obtained from the TAIR8 cDNA set using in each case the first cDNA model (ATNG00000.1). To compare this set of single copy genes to the 454 transcriptome data, an analysis was carried out by running the BLASTALL program version 2.2.16 [83] in the UNIX environment of an Apple Powerbook Pro. The 956 sequences were BLASTed against a database made of all the 454 sequence reads. Alignment results with an E value > 10^-11 ^were saved and parsed to eliminate reads that had single instances of SNP or indels and to rank the genes according to the number of read hits. The six genes that aligned to more than sixty reads were examined to identify "haplotypes" indicative of two or more copies.

### Genome size estimation

*Camelina *lines (Additional File 8) were grown in the greenhouse at temperatures fluctuating between 16°C and 26°C with 16 hour day length supplemented by halogen lights. The nuclei were extracted from leaves according to Henry *et al *[74]. Nuclei were also extracted from approximately 50 seeds of all species, except *C. laxa *and *C. hispida*, which are late flowering. The seeds were crushed with a pestle in 1.4 mL of the same extraction buffer used for the leaves. The fluid was then drawn through four layers of cheesecloth and strained and processed as for the leaf nuclei. Nuclei of diploid and tetraploids of *Arabidopsis thaliana *accession Col-0 (1 C genome size 157 Mb and 314 Mb, respectively [75]), and tetraploid *Arabidopsis arenosa *accession Care-1 (1C genome size 480 Mb [Dilkes, unpublished results]) were used as standards for DNA content. Data was collected on two different days and normalized separately to account for daily fluctuations in flow cytometer performance. The 2C, 4C, and 8C nuclear peaks were used in a regression analysis of measured fluorescence intensity versus nuclear DNA content, producing equations of genome size versus fluorescence that were used to estimate the 2C content of *Camelina *nuclei.

### Phylogenetic inference

*FAD2 *and *FAE1 *were PCR amplified from several *Camelina *species and outgroups (Additional File 8) using primers designed from *C. sativa FAD2 *and *FAE1 *sequences (Additional File 1). Amplified fragments for *FAD2 *and *FAE1 *were cloned as described for *C. sativa *above, then aligned by translated amino acid sequences using MacClade 4.05 [84]. ModelTest 3.7 [58] in PAUP* 4.0 b [59] was used to determine the model of sequence evolution favored by the data for each gene. Subsequent maximum likelihood (ML) analyses were performed in PAUP* 4.0 b using a heuristic search with tree bisection reconnection (TBR) branch swapping. ML clade support using 100 bootstrap data sets were assessed and this support is presented on the most likely tree recovered from the ML heuristic search.

### Accession numbers

*FAD2 *and *FAE1 *sequences from *Camelina *species and outgroups have been deposited in Genbank at the NCBI [Genbank: GU929417 - GU929441].

## Authors' contributions

CH carried out the amplification of *C. sativa *and *Camelinae *genomic sequences, participated in the sequence alignment, and helped draft the manuscript. RFD carried out the Southern blot analysis and the amplification of *C. sativa *genomic sequences, participated in the sequence alignment, and helped draft the manuscript. MB carried out the phylogenetic analyses and helped draft the manuscript. LC participated in the design and analysis of the study, analyzed the 454 transcriptome data, and helped draft the manuscript. JS carried out the qPCR analyses. EG carried out the flow cytometry analysis. CKS participated in the sequence alignment, in the design of the study, and helped draft the manuscript. TN, JD, and JK conceived of the study. All authors read and approved the final manuscript.

## Supplementary Material

Additional file 1 **Primers used for amplification of genomic regions of *C. sativa***. Table of primers used in the amplification of genomic regions of *Camelina sativa* Click here for file

Additional file 2 ***FAD2 *and *FAE1 *nucleotide alignments**. (A) Nucleotide sequence comparison of the three *Camelina sativa FAD2 *sequences and the *Arabidopsis thaliana FAD2 *sequence [Genbank: NM_112047]. Green underlines indicate the start and stop codons, the blue underline indicates the BamHI site in *CsFAD2 *A and *AtFAD2*, the orange underline indicates the ER localization signal, and the grey underline indicates the glutamine at amino acid position 44. The three His boxes described by Tocher *et al *[44] are indicated with red boxes. (B) Nucleotide sequence comparison of the three *Camelina sativa FAE1 *sequences and the *Arabidopsis thaliana FAE1 *sequence [Genbank: NM_119617]. Green underlines indicate the start and stop codons. Blue underlines below the sequence indicate the asparagine at amino acid position 424 and the highly conserved histidine and cysteine residues described by Ghanevati and Jaworski [49,50]. The red box indicates the region highly conserved among condensing enzymes in very long chain fatty acid biosynthesis [62] Click here for file

Additional file 3 **Camelineae FAD2 and FAE1 protein alignment**. (A) Amino acid sequence comparison of FAD2 sequences from species in the tribe Camelineae. The amino acid at position 44 is indicated with a blue underline while the green underline indicates the ER localization signal [45]. The three His boxes described by Tocher *et al *[44] are indicated with red boxes. The *Arabidopsis thaliana *FAD2 sequence was obtained from Genbank [Genbank:NP_187819]. (B) Amino acid sequence comparison of FAE1 sequences from species in the tribe Camelineae. Blue underlines below the sequence indicate the asparagine at amino acid position 424 and the highly conserved histidine and cysteine residues described by Ghanevati and Jaworski [49,50]. The red box indicates the region highly conserved among condensing enzymes in very long chain fatty acid biosynthesis [62]. The *Arabidopsis thaliana *FAE1 sequence was obtained from Genbank [Genbank:NP_195178]. Click here for file

Additional file 4 **Primers used for qPCR analyses**. List of primers used for qPCR analyses Click here for file

Additional file 5 **SNPs distinguishing each copy of *CsFAD2 *and *CsFAE1***. List of SNPs used in Sequenom MassARRAY™ analyses to distinguish the three copies of *CsFAD2 *and of *CsFAE1* Click here for file

Additional file 6 **Dot plots of KCS17-FAE1 intergenic region**. Sequences obtained for *CsKCS17-FAE1A*, *B *and *C *were aligned with each other and with Arabidopsis orthologous region two at a time in a dot plot with parameters set for perfect conservation on a sliding window of 9 bases. Click here for file

Additional file 7 **Deep sequencing reads for 6 predicted single-copy genes in *C. sativa***. Sequences determined by 454 sequencing of cDNA from 15 DPA *C. sativa *seeds, aligned with 6 genes predicted by Duarte *et al *[35] to be single-copy in flowering plants. Click here for file

Additional file 8 **Plant species and sources**. List of plant species used and their sources. Click here for file
