# Supplementary material for: Polyploid genome of Camelina sativa revealed by isolation of fatty acid synthesis genes
Source: BMC Plant Biol. 2010 Oct 27;10:233. doi: 10.1186/1471-2229-10-233 (PMC3017853; doi:10.1186/1471-2229-10-233)
Supplement: Additional file 1 — Primers used for amplification of genomic regions of C. sativa. Table of primers used in the amplification of genomic regions of Camelina sativa [file 1471-2229-10-233-S1.DOCX]

## Additional File 1 - Primers used for amplification of genomic regions of *C. sativa*

| **Application** | **Primer Name** | **Sequence Source** | **Primer sequence (5’ – 3’ )** |
| --- | --- | --- | --- |
| Southern analysis of FAD2 | FAD2_631F | *Arabidopsis thaliana* | TCAACAACCCTCTTGGACGCATCA |
|  | FAD2_832R | *Arabidopsis thaliana* | CTTGTGCAGCAGCGTAACGGTAAA |
| Southern analysis of FAE1 | AtFAE1 probe F | *Arabidopsis thaliana* | AGACGGTCCAAGTACAAGCTAGTTC |
|  | AtFAE1 probe R | *Arabidopsis thaliana* | CCAAATCTATGTAACGTTGATCT |
| Southern analysis of LFY | AtLFY probe F | *Arabidopsis thaliana* | GATGCGGCGGGGAATAACGGCGGAG |
|  | AtLFY probe R | *Arabidopsis thaliana* | CCTGAAGAAGGAACTCACGGCATT |
| Cloning of CsFAD2 coding region | AtFAD2_start | *Arabidopsis thaliana* | AACATGGGTGCAGGTGGAAGAATG |
|  | AtFAD2_stop2 | *Arabidopsis thaliana* | TCATAACTTATTGTTGTACCAGTAC |
| Cloning of CsFAE1 coding region | CaFAE1 start | *Crambe abyssinica* | ATGACGTCCATTAACGTAAAGCTC |
|  | CaFAE1 stop | *Crambe abyssinica* | TTAGGACCGACCGTTTTGGGC |
| RACE PCR of CsFAD2A 5’ utr | FAD2_5’race2 | *Camelina sativa* | GGGATCCTGTGTTGGAATGGTGACGGAC |
| Cloning of CsFAD2 5’ intron | CsFAD2 5’ utr F | *Camelina sativa* | GTGGAGGAGCTTCTTCCTCGTAGG |
|  | CsFAD2 166R | *Camelina sativa* | GGAGAAAGAGCGAGGGATAGAGCG |
| CsKCS17-CsFAE1 intergenic region “A” and “C” (initial clones) | AtKCS F | *Arabidopsis thaliana* | GGGTGGCTCTTCGCAATGTCGAGCCC |
|  | CsFAE1 5’ RACE | *Camelina sativa* | GAGGCTTTTCCGGCAAGTAACGCCG |
| CsKCS17-CsFAE1 intergenic region “A” | AtKCS cons F | *Arabidopsis thaliana* | GGTATGAATTGGCTTACACGGAAG |
|  |  |  |  |
|  | CsFAE1A_R2 | *Camelina sativa* | TATATTGCCAATATAAGTATTAAAGGTCC |
| CsKCS17-CsFAE intergenic region “B” | AtKCS cons F | *Arabidopsis thaliana* | GGTATGAATTGGCTTACACGGAAG |
|  | CsFAE1B_R | *Camelina sativa* | TATATTGCCAATATAAGTATTAAAGGTCC |
| CsKCS17-CsFAE intergenic region “C” | AtKCS cons F | *Arabidopsis thaliana* | GGTATGAATTGGCTTACACGGAAG |
|  | CsFAE1C_R | *Camelina sativa* | GGTAGAGATCGTTTGTGGTAAGCG |
| Camelinae FAD2 | CsFAD2 start | *Camelina sativa* | ATGGGTGCAGGTGGAAGAATGC |
|  | CsFAD2 stop | *Camelina sativa* | TCATAACTTATTGTTGTACCAGTACACACC |
| Camelinae FAE1 | CsFAE1 start | *Camelina sativa* | ATGACGTCCGTTAACGCAAAGCTC |
|  | CsFAE1 stop | *Camelina sativa* | TTAGGACCGACCGTTTTTGACATG |
