# Supplementary material for: Polyploid genome of Camelina sativa revealed by isolation of fatty acid synthesis genes
Source: BMC Plant Biol. 2010 Oct 27;10:233. doi: 10.1186/1471-2229-10-233 (PMC3017853; doi:10.1186/1471-2229-10-233)
Supplement: Additional file 2 — FAD2 and FAE1 nucleotide alignments. (A) Nucleotide sequence comparison of the three Camelina sativa FAD2 sequences and the Arabidopsis thaliana FAD2 sequence [Genbank: NM_112047]. Green underlines indicate the start and stop codons, the blue underline indicates the BamHI site in CsFAD2 A and AtFAD2, the orange underline indicates the ER localization signal, and the grey underline indicates the glutamine at amino acid position 44. The three His boxes described by Tocher et al [44] are indicated with red boxes. (B) Nucleotide sequence comparison of the three Camelina sativa FAE1 sequences and the Arabidopsis thaliana FAE1 sequence [Genbank: NM_119617]. Green underlines indicate the start and stop codons. Blue underlines below the sequence indicate the asparagine at amino acid position 424 and the highly conserved histidine and cysteine residues described by Ghanevati and Jaworski [49,50]. The red box indicates the region highly conserved among condensing enzymes in very long chain fatty acid biosynthesis [62] [file 1471-2229-10-233-S2.PDF]

A

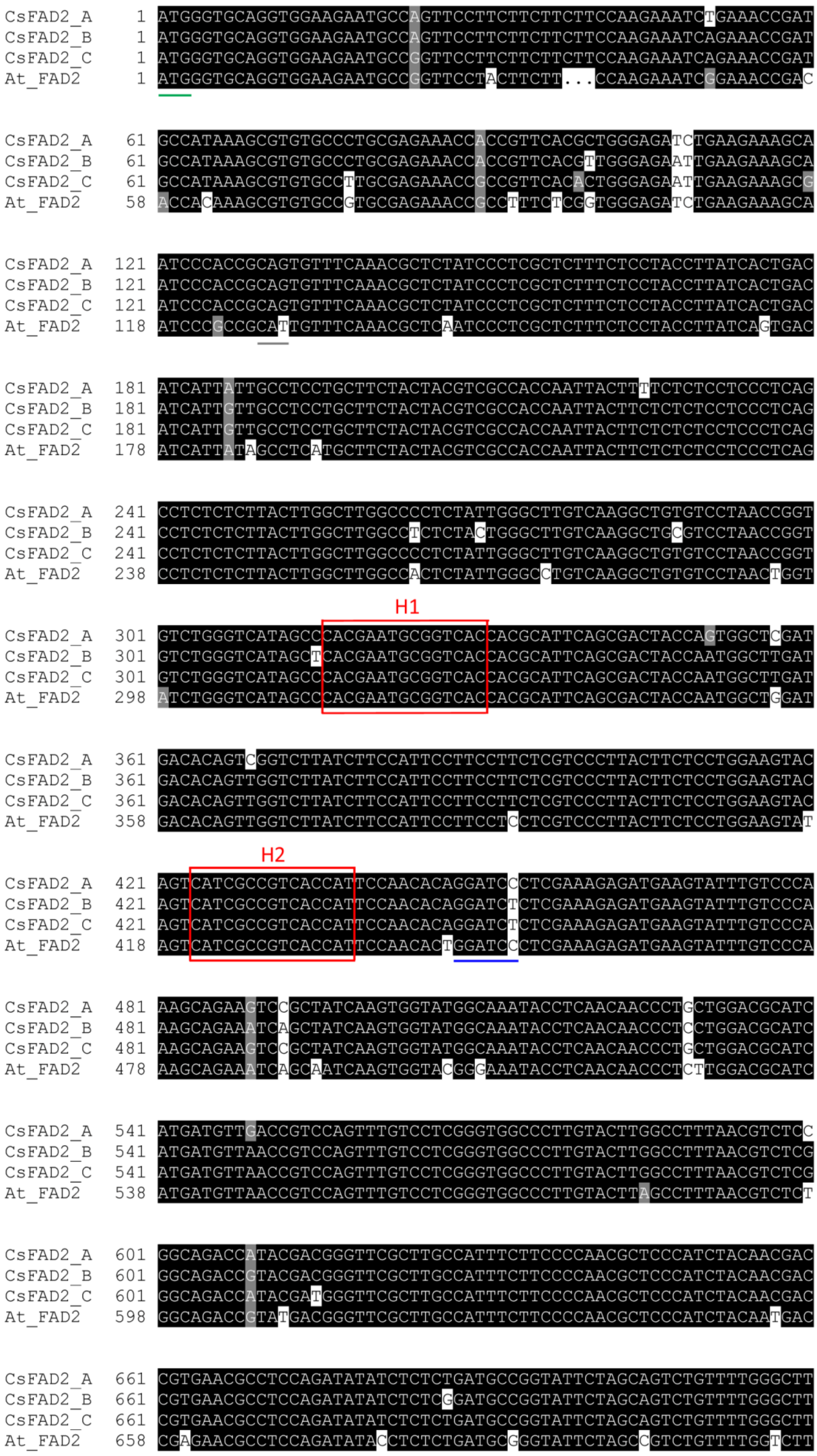
CsFAD2_A 1 ATGGGTGCAGGTGGAAGAATGCCAGTTCCTTCTTCTTCTTCCAAGAAATCTGAAACCGAT
CsFAD2_B 1 ATGGGTGCAGGTGGAAGAATGCCAGTTCCTTCTTCTTCTTCCAAGAAATCAGAAACCGAT
CsFAD2_C 1 ATGGGTGCAGGTGGAAGAATGCCGGTTCCTTCTTCTTCTTCCAAGAAATCAGAAACCGAT
At_FAD2 1 ATGGGTGCAGGTGGAAGAATGCCGGTTCCTACTTCTT...CCAAGAAATCGGAAACCGAC
CsFAD2_A 61 GCCATAAAGCGTGTGCCCTGCGAGAAACCACCGTTCACGCTGGGAGATCTGAAGAAAGCA
CsFAD2_B 61 GCCATAAAGCGTGTGCCCTGCGAGAAACCACCGTTCACGTTGGGAGAATTGAAGAAAGCA
CsFAD2_C 61 GCCATAAAGCGTGTGCCTTGCGAGAAACCGCCGTTCACACTGGGAGAATTGAAGAAAGCG
At_FAD2 58 ACCACAAAGCGTGTGCCGTGCGAGAAACCGCCTTTCTCGGTGGGAGATCTGAAGAAAGCA
CsFAD2_A 121 ATCCCACCGCAGTGTTTCAAACGCTCTATCCCTCGCTCTTTCTCCTACCTTATCACTGAC
CsFAD2_B 121 ATCCCACCGCAGTGTTTCAAACGCTCTATCCCTCGCTCTTTCTCCTACCTTATCACTGAC
CsFAD2_C 121 ATCCCACCGCAGTGTTTCAAACGCTCTATCCCTCGCTCTTTCTCCTACCTTATCACTGAC
At_FAD2 118 ATCCCGCCGCATTGTTTCAAACGCTCAATCCCTCGCTCTTTCTCCTACCTTATCAGTGAC
CsFAD2_A 181 ATCATTATTGCCTCCTGCTTCTACTACGTCGCCACCAATTACTTTTCTCTCCTCCCTCAG
CsFAD2_B 181 ATCATTGTTGCCTCCTGCTTCTACTACGTCGCCACCAATTACTTCTCTCTCCTCCCTCAG
CsFAD2_C 181 ATCATTGTTGCCTCCTGCTTCTACTACGTCGCCACCAATTACTTCTCTCTCCTCCCTCAG
At_FAD2 178 ATCATTATAGCCTCATGCTTCTACTACGTCGCCACCAATTACTTCTCTCTCCTCCCTCAG
CsFAD2_A 241 CCTCTCTCTTACTTGGCTTGGCCCCTCTATTGGGCTTGTCAAGGCTGTGTCCTAACCGGT
CsFAD2_B 241 CCTCTCTCTTACTTGGCTTGGCCTCTCTACTGGGCTTGTCAAGGCTGCGTCCTAACCGGT
CsFAD2_C 241 CCTCTCTCTTACTTGGCTTGGCCCCTCTATTGGGCTTGTCAAGGCTGTGTCCTAACCGGT
At_FAD2 238 CCTCTCTCTTACTTGGCTTGGCCACTCTATTGGGCCTGTCAAGGCTGTGTCCTAACTGGT
H1
CsFAD2_A 301 GTCTGGGTCATAGCCCACGAATGCGGTCACCACGCATTCAGCGACTACCAGTGGCTCGAT
CsFAD2_B 301 GTCTGGGTCATAGCTCACGAATGCGGTCACCACGCATTCAGCGACTACCAATGGCTTGAT
CsFAD2_C 301 GTCTGGGTCATAGCCCACGAATGCGGTCACCACGCATTCAGCGACTACCAATGGCTTGAT
At_FAD2 298 ATCTGGGTCATAGCCCACGAATGCGGTCACCACGCATTCAGCGACTACCAATGGCTGGAT
CsFAD2_A 361 GACACAGTCGGTCTTATCTTCCATTCCTTCCTTCTCGTCCCTTACTTCTCCTGGAAGTAC
CsFAD2_B 361 GACACAGTTGGTCTTATCTTCCATTCCTTCCTTCTCGTCCCTTACTTCTCCTGGAAGTAC
CsFAD2_C 361 GACACAGTTGGTCTTATCTTCCATTCCTTCCTTCTCGTCCCTTACTTCTCCTGGAAGTAC
At_FAD2 358 GACACAGTTGGTCTTATCTTCCATTCCTTCCTCCTCGTCCCTTACTTCTCCTGGAAGTAT
H2
CsFAD2_A 421 AGTCATCGCCGTCACCATTCCAACACAGGATCCCTCGAAAGAGATGAAGTATTTGTCCCA
CsFAD2_B 421 AGTCATCGCCGTCACCATTCCAACACAGGATCTCTCGAAAGAGATGAAGTATTTGTCCCA
CsFAD2_C 421 AGTCATCGCCGTCACCATTCCAACACAGGATCTCTCGAAAGAGATGAAGTATTTGTCCCA
At_FAD2 418 AGTCATCGCCGTCACCATTCCAACACTGGATCCCTCGAAAGAGATGAAGTATTTGTCCCA
CsFAD2_A 481 AAGCAGAAGTCCGCTATCAAGTGGTATGGCAAATACCTCAACAACCCTGCTGGACGCATC
CsFAD2_B 481 AAGCAGAAATCAGCTATCAAGTGGTATGGCAAATACCTCAACAACCCTCCTGGACGCATC
CsFAD2_C 481 AAGCAGAAGTCCGCTATCAAGTGGTATGGCAAATACCTCAACAACCCTGCTGGACGCATC
At_FAD2 478 AAGCAGAAATCAGCAATCAAGTGGTACGGGAAATACCTCAACAACCCTCTTGGACGCATC
CsFAD2_A 541 ATGATGTTGACCGTCCAGTTTGTCCTCGGGTGGCCCTTGTACTTGGCCTTTAACGTCTCC
CsFAD2_B 541 ATGATGTTAACCGTCCAGTTTGTCCTCGGGTGGCCCTTGTACTTGGCCTTTAACGTCTCG
CsFAD2_C 541 ATGATGTTAACCGTCCAGTTTGTCCTCGGGTGGCCCTTGTACTTGGCCTTTAACGTCTCG
At_FAD2 538 ATGATGTTAACCGTCCAGTTTGTCCTCGGGTGGCCCTTGTACTTAGCCTTTAACGTCTCT
CsFAD2_A 601 GGCAGACCATACGACGGGTTCGCTTGCCATTTCTTCCCCAACGCTCCCATCTACAACGAC
CsFAD2_B 601 GGCAGACCGTACGACGGGTTCGCTTGCCATTTCTTCCCCAACGCTCCCATCTACAACGAC
CsFAD2_C 601 GGCAGACCATACGATGGGTTCGCTTGCCATTTCTTCCCCAACGCTCCCATCTACAACGAC
At_FAD2 598 GGCAGACCGTATGACGGGTTCGCTTGCCATTTCTTCCCCAACGCTCCCATCTACAATGAC
CsFAD2_A 661 CGTGAACGCCTCCAGATATATCTCTCTGATGCCGGTATTCTAGCAGTCTGTTTTGGGCTT
CsFAD2_B 661 CGTGAACGCCTCCAGATATATCTCTCGGATGCCGGTATTCTAGCAGTCTGTTTTGGGCTT
CsFAD2_C 661 CGTGAACGCCTCCAGATATATCTCTCTGATGCCGGTATTCTAGCAGTCTGTTTTGGGCTT
At_FAD2 658 CGAGAACGCCTCCAGATATACCTCTCTGATGCGGGTATTCTAGCCGTCTGTTTTGGTCTT

```
CsFAD2_A  721 TACCGTTACGCCGCTGCACAAGGATTGGCCTCGATGATCTGCCTCTACGGAGTACCACTT
CsFAD2_B  721 TACCGTTACGCTGCTGCACAAGGAATGGCCTCGATGATCTGCCTCTACGGAGTACCGCTT
CsFAD2_C  721 TACCGTTACGCCGCTGCACAAGGATTGGCCTCGATGATCTGCCTCTACGGAGTACCACTT
At_FAD2   718 TACCGTTACGCTGCTGCACAAGGGATGGCCTCGATGATCTGCCTCTACGGAGTACCGCTT

CsFAD2_A  781 CTGATAGTGAACGCGTTCCTCGTCTTGATCACTTACTTGCAGCACACTCATCCTGCGTTG
CsFAD2_B  781 CTGATAGTGAACGCGTTCCTCGTCTTGATCACTTACTTGCAGCACACTCATCCTGCGTTG
CsFAD2_C  781 CTGATAGTAAACGCGTTCCTCGTCTTGATCACTTACTTGCAGCACACTCATCCTGCGTTG
At_FAD2   778 CTGATAGTGAATGCGTTCCTCGTCTTGATCACTTACTTGCAGCACACTCATCCCTCGTTG

CsFAD2_A  841 CCTCACTACGATTCATCCGAGTGGGATTGGCTTAGGGGAGCTTTGGCTACCGTAGACAGA
CsFAD2_B  841 CCTCACTACGATTCATCCGAGTGGGATTGGCTTAGGGGAGCTTTGGCTACCGTTGACAGA
CsFAD2_C  841 CCTCACTACGATTCATCCGAGTGGGATTGGCTTAGGGGAGCTTTGGCTACCGTAGACAGA
At_FAD2   838 CCTCACTACGATTCATCAGAGTGGGACTGGCTCAGGGGAGCTTTGGCTACCGTAGACAGA

                                                                  H3
CsFAD2_A  901 GACTATGGAATCTTGAATAAGGTGTTCCACAACATCACGGACACACATGTGGCTCATCAT
CsFAD2_B  901 GACTATGGAATCTTGAACAAGGTGTTCCACAACATCACGGACACACATGTGGCTCATCAT
CsFAD2_C  901 GACTATGGAATCTTGAACAAGGTGTTCCACAACATCACGGACACACATGTGGCTCATCAT
At_FAD2   898 GACTACGGAATCTTGAACAAGGTGTTCCACAACATTACAGACACACACGTGGCTCATCAC

CsFAD2_A  961 CTGTTCTCGACAATGCCGCATTATAATGCGATGGAAGCTACAAAGGCGATAAAGCCAATA
CsFAD2_B  961 CTGTTCTCGACAATGCCACATTATAATGCGATGGAAGCTACAAAGGCGATAAAGCCAATA
CsFAD2_C  961 CTGTTTTCGACAATGCCGCATTATAATGCGATGGAAGCTACAAAGGCGATAAAGCCAATA
At_FAD2   958 CTGTTCTCGACAATGCCGCATTATAACGCAATGGAAGCTACAAAGGCGATAAAGCCAATT

CsFAD2_A 1021 CTCGGTGACTATTACCAGTTCGACGGAACACCATGGTATGTGGCCATGTATAGGGAGGCA
CsFAD2_B 1021 CTCGGTGACTACTACCAGTTCGACGGAACACCGTGGTATGTGGCGATGTATAGGGAGGCA
CsFAD2_C 1021 CTCGGTGACTATTACCAGTTCGACGGAACACCATGGTATATGGCCATGTATAGGGAGGCA
At_FAD2  1018 CTGGGAGACTATTACCAGTTCGATGGAACACCGTGGTATGTAGCGATGTATAGGGAGGCA

CsFAD2_A 1081 AAGGAGTGTATCTATGTAGAACCGGACAGGGAAGGTGACAAGAAAGGTGTGTACTGGTAC
CsFAD2_B 1081 AAGGAGTGTATCTATGTAGAACCGGACAGAGAAGGTGACAAGAAAGGTGTGTACTGGTAC
CsFAD2_C 1081 AAGGAGTGTATCTATGTAGAACCGGACAGGGAAGGTGACAAGAAAGGTGTGTACTGGTAC
At_FAD2  1078 AAGGAGTGTATCTATGTAGAACCGGACAGGGAAGGTGACAAGAAAGGTGTGTACTGGTAC

CsFAD2_A 1141 AACAATAAGTTATGA
CsFAD2_B 1141 AACAATAAGTTATGA
CsFAD2_C 1141 AACAATAAGTTATGA
At_FAD2  1138 AACAATAAGTTATGA
```

B

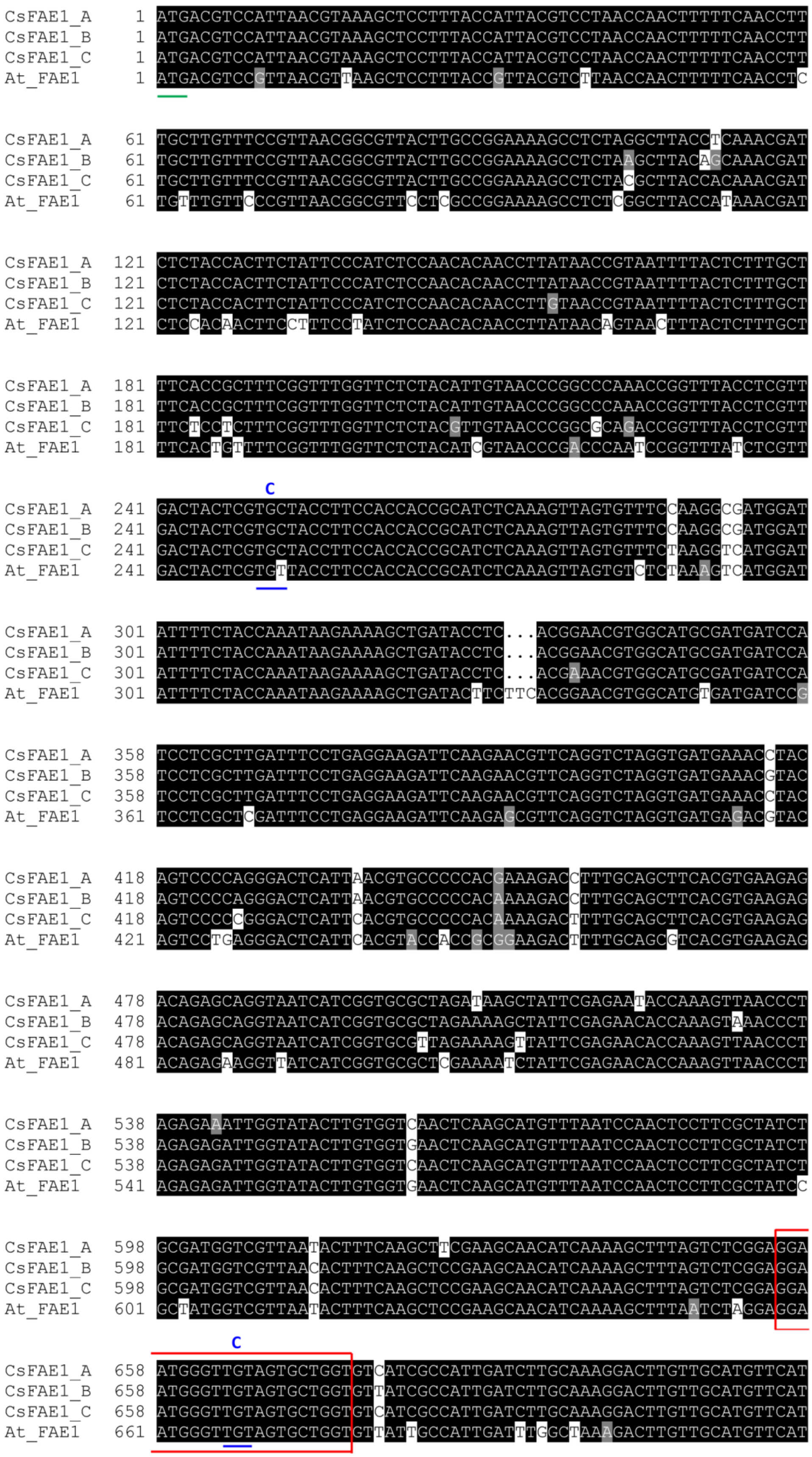
CsFAE1_A 1 ATGACGTCCATTAACGTAAAGCTCCTTTACCATTACGTCCTAACCAACTTTTTCAACCTT
CsFAE1_B 1 ATGACGTCCATTAACGTAAAGCTCCTTTACCATTACGTCCTAACCAACTTTTTCAACCTT
CsFAE1_C 1 ATGACGTCCATTAACGTAAAGCTCCTTTACCATTACGTCCTAACCAACTTTTTCAACCTT
At_FAE1 1 ATGACGTCCGTTAACGTTAAGCTCCTTTACCGTTACGTCTTAACCAACTTTTTCAACCTC
CsFAE1_A 61 TGCTTGTTTCCGTTAACGGCGTTACTTGCCGGAAAAGCCTCTAGGCTTACCTCAAACGAT
CsFAE1_B 61 TGCTTGTTTCCGTTAACGGCGTTACTTGCCGGAAAAGCCTCTAAGCTTACAGCAAACGAT
CsFAE1_C 61 TGCTTGTTTCCGTTAACGGCGTTACTTGCCGGAAAAGCCTCTACGCTTACCACAAACGAT
At_FAE1 61 TGTTTGTTCCCGTTAACGGCGTTCCTCGCCGGAAAAGCCTCTCGGCTTACCATAAACGAT
CsFAE1_A 121 CTCTACCACTTCTATTCCCATCTCCAACACAACCTTATAACCGTAATTTTACTCTTTGCT
CsFAE1_B 121 CTCTACCACTTCTATTCCCATCTCCAACACAACCTTATAACCGTAATTTTACTCTTTGCT
CsFAE1_C 121 CTCTACCACTTCTATTCCCATCTCCAACACAACCTTGTAACCGTAATTTTACTCTTTGCT
At_FAE1 121 CTCCACAACTTCCTTTCCTATCTCCAACACAACCTTATAACAGTAACTTTACTCTTTGCT
CsFAE1_A 181 TTCACCGCTTTCGGTTTGGTTCTCTACATTGTAACCCGGCCCAAACCGGTTTACCTCGTT
CsFAE1_B 181 TTCACCGCTTTCGGTTTGGTTCTCTACATTGTAACCCGGCCCAAACCGGTTTACCTCGTT
CsFAE1_C 181 TTCTCCTCTTTCGGTTTGGTTCTCTACGTTGTAACCCGGCGCAGACCGGTTTACCTCGTT
At_FAE1 181 TTCACTGTTTTCGGTTTGGTTCTCTACATCGTAACCCGACCCAATCCGGTTTATCTCGTT
C
CsFAE1_A 241 GACTACTCGTGCTACCTTCCACCACCGCATCTCAAAGTTAGTGTTTCCAAGGCGATGGAT
CsFAE1_B 241 GACTACTCGTGCTACCTTCCACCACCGCATCTCAAAGTTAGTGTTTCCAAGGCGATGGAT
CsFAE1_C 241 GACTACTCGTGCTACCTTCCACCACCGCATCTCAAAGTTAGTGTTTCTAAGGTCATGGAT
At_FAE1 241 GACTACTCGTGTTACCTTCCACCACCGCATCTCAAAGTTAGTGTCTCTAAAGTCATGGAT
CsFAE1_A 301 ATTTTCTACCAAATAAGAAAAGCTGATACCTC...ACGGAACGTGGCATGCGATGATCCA
CsFAE1_B 301 ATTTTCTACCAAATAAGAAAAGCTGATACCTC...ACGGAACGTGGCATGCGATGATCCA
CsFAE1_C 301 ATTTTCTACCAAATAAGAAAAGCTGATACCTC...ACGAAACGTGGCATGCGATGATCCA
At_FAE1 301 ATTTTCTACCAAATAAGAAAAGCTGATACTTCTTCACGGAACGTGGCATGTGATGATCCG
CsFAE1_A 358 TCCTCGCTTGATTTCCTGAGGAAGATTCAAGAACGTTCAGGTCTAGGTGATGAAACCTAC
CsFAE1_B 358 TCCTCGCTTGATTTCCTGAGGAAGATTCAAGAACGTTCAGGTCTAGGTGATGAAACGTAC
CsFAE1_C 358 TCCTCGCTTGATTTCCTGAGGAAGATTCAAGAACGTTCAGGTCTAGGTGATGAAACCTAC
At_FAE1 361 TCCTCGCTCGATTTCCTGAGGAAGATTCAAGAGCGTTCAGGTCTAGGTGATGAGACGTAC
CsFAE1_A 418 AGTCCCCAGGGACTCATTAACGTGCCCCCACGAAAGACCTTTGCAGCTTCACGTGAAGAG
CsFAE1_B 418 AGTCCCCAGGGACTCATTAACGTGCCCCCACAAAAGACCTTTGCAGCTTCACGTGAAGAG
CsFAE1_C 418 AGTCCCCCGGGACTCATTCACGTGCCCCCACAAAAGACTTTTGCAGCTTCACGTGAAGAG
At_FAE1 421 AGTCCTGAGGGACTCATTCACGTACCACCGCGGAAGACTTTTGCAGCGTCACGTGAAGAG
CsFAE1_A 478 ACAGAGCAGGTAATCATCGGTGCGCTAGATAAGCTATTCGAGAATACCAAAGTTAACCCT
CsFAE1_B 478 ACAGAGCAGGTAATCATCGGTGCGCTAGAAAAGCTATTCGAGAACACCAAAGTAAACCCT
CsFAE1_C 478 ACAGAGCAGGTAATCATCGGTGCGTTAGAAAAGTTATTCGAGAACACCAAAGTTAACCCT
At_FAE1 481 ACAGAGAAGGTTATCATCGGTGCGCTCGAAAATCTATTCGAGAACACCAAAGTTAACCCT
CsFAE1_A 538 AGAGAAATTGGTATACTTGTGGTCAACTCAAGCATGTTTAATCCAACTCCTTCGCTATCT
CsFAE1_B 538 AGAGAGATTGGTATACTTGTGGTGAACTCAAGCATGTTTAATCCAACTCCTTCGCTATCT
CsFAE1_C 538 AGAGAGATTGGTATACTTGTGGTCAACTCAAGCATGTTTAATCCAACTCCTTCGCTATCT
At_FAE1 541 AGAGAGATTGGTATACTTGTGGTGAACTCAAGCATGTTTAATCCAACTCCTTCGCTATCC
CsFAE1_A 598 GCGATGGTCGTTAATACTTTCAAGCTTCGAAGCAACATCAAAAGCTTTAGTCTCGGAGGA
CsFAE1_B 598 GCGATGGTCGTTAACACTTTCAAGCTCCGAAGCAACATCAAAAGCTTTAGTCTCGGAGGA
CsFAE1_C 598 GCGATGGTCGTTAACACTTTCAAGCTCCGAAGCAACATCAAAAGCTTTAGTCTCGGAGGA
At_FAE1 601 GCTATGGTCGTTAATACTTTCAAGCTCCGAAGCAACATCAAAAGCTTTAATCTAGGAGGA
C
CsFAE1_A 658 ATGGGTTGTAGTGCTGGTGTCATCGCCATTGATCTTGCAAAGGACTTGTTGCATGTTCAT
CsFAE1_B 658 ATGGGTTGTAGTGCTGGTGTTATCGCCATTGATCTTGCAAAGGACTTGTTGCATGTTCAT
CsFAE1_C 658 ATGGGTTGTAGTGCTGGTGTCATCGCCATTGATCTTGCAAAGGACTTGTTGCATGTTCAT
At_FAE1 661 ATGGGTTGTAGTGCTGGTGTTATTGCCATTGATTTGGCTAAAGACTTGTTGCATGTTCAT

```
CsFAE1_A  718 AAAAACACTTATGCACTTGTGGTGAGCACTGAGAACATCACTCAAGGCATTTATGCTGGC
CsFAE1_B  718 AAAAACACTTATGCACTTGTGGTGAGCACTGAGAACATCACTCAAGGCATTTATGCTGGC
CsFAE1_C  718 AAACACACTTATGCACTTGTGGTGAGCACTGAGAACATCACTCAAGGCATTTATGCTGGC
At_FAE1   721 AAAAACACTTATGCTCTTGTGGTGAGCACTGAGAACATCACACAAGGCATTTATGCTGGA

                                          C
CsFAE1_A  778 GAAAATAGATCCATGATGGTTAGCAATTGCTTGTTCCGTGTTGGTGGCGCAGCGATTTTG
CsFAE1_B  778 GAAAACAGATCCATGATGGTTAGCAATTGCTTGTTTCGTGTTGGTGGGGCAGCGATTTTG
CsFAE1_C  778 GAAAATAGATCCATGATGGTTAGCAATTGCTTGTTTCGTGTTGGTGGGGCAGCGATTTTG
At_FAE1   781 GAAAATAGATCAATGATGGTTAGCAATTGCTTGTTTCGTGTTGGTGGGGCCGCGATTTTG

CsFAE1_A  838 CTCTCCAACAAGCCAGGAGATCGGAGACGGTCCAAGTACAAGTTATGTCATACTGTTCGA
CsFAE1_B  838 CTCTCCAACAAACTGGGAGATCGGAGACGGTCCAAGTACAAGCTATGTCATACTGTTCGA
CsFAE1_C  838 CTATCCAACAAGCCGGGAGATCGGAGACGGTCCAAGTACAAGCTATGTCACACGGTTCGG
At_FAE1   841 CTCTCTAACAAGTCGGGAGACCGGAGACGGTCCAAGTACAAGCTAGTTCACACGGTCCGA

                  H                             C
CsFAE1_A  898 ACGCATACCGGAGCTGATGACATGTCTTTTCGATGTGTGCAACAAGGAGACGATGAGAGC
CsFAE1_B  898 ACGCATACCGGAGCTGATGACAAGTCTTTTCGATGTGTGCAACAAGGAGACGATGAGGGC
CsFAE1_C  898 ACTCATACCGGAGCTGATGACAAGTCTTTTCGATGTGTGCAACAAGGAGACGATGAGAGC
At_FAE1   901 ACGCATACTGGAGCTGATGACAAGTCTTTTCGATGTGTGCAACAAGAAGACGATGAGAGC

CsFAE1_A  958 GGTAAAATCGGAGTTTGTCTGTCAAAGGACATAACCGTTGTTGCGGGATAGCGCTTAAG
CsFAE1_B  958 GGTAAAATCGGAGTTTGTCTGTCAAAGGACATAACCGTTGTTGCGGGACAGCGCTTAAG
CsFAE1_C  958 GGTAAAATCGGAGTTTGTCTGTCAAAGGACATAACAGTTGTTGCGGGACAGCGCTTAAG
At_FAE1   961 GGCAAAATCGGAGTTTGTCTGTCAAAGGACATAACCAATGTTGCGGGACAACACTTACG

CsFAE1_A 1018 AAAAACATAGCAACGTTGGGTCCGTTGATTCTTCCTTTAAGCGAAAAATTTCTGTTTTTA
CsFAE1_B 1018 AAAAACATAGCAACGTTGGGTCCGTTGATTCTTCCTTTAAGCGAAAAGTTTCTGTTTTTA
CsFAE1_C 1018 AAAAACATAGCAACGTTAGGTCCGTTGATTCTTCCTTTAAGCGAAAAGTTTCTGTTCTTA
At_FAE1  1021 AAAAATATAGCAACATTGGGTCCGTTGATTCTTCCTTTAAGCGAAAAGTTTCTTTTTTTC

CsFAE1_A 1078 GTAACCTTCATCGCCAAGAAACTTTTGAAGGACAAGATCAAGCACTATTACGTCCCGGAT
CsFAE1_B 1078 GTTACCTTCATCGCCAAGAAACTTTTGAAGGACAAGATCAAGCACTGTTACGTCCCGGAT
CsFAE1_C 1078 GTTACCTTCATCGCCAAGAAACTTTTGAAGGACAAGATCAAGCACTATTACGTCCCGGAT
At_FAE1  1081 GCTACCTTCGTCGCCAAGAAACTTCTAAAGGATAAAATCAAGCATTACTATGTTCCGGAT

                                 H     C     H
CsFAE1_A 1138 TTCAAGCTTGCTATTGACCATTTCTGTATTCATGCGGGAGGCAGAGCCGTGATCGATGTG
CsFAE1_B 1138 TTCAAGCTTGCTATCGACCATTTCTGTATTCATGCGGGAGGCAGAGCCGTGATCGATGTG
CsFAE1_C 1138 TTCAAGCTTGCTATTGACCATTTCTGTATTCATGCCGGAGGCAGAGCCGTAATCGATGTG
At_FAE1  1141 TTCAAGCTTGCTGTTGACCATTTCTGTATTCATGCCGGAGGCAGAGCCGTGATCGATGAG

                                                                        H
CsFAE1_A 1198 CTTGAGAAGAGCTTAGGACTATCTCCAATCGATGTGGAGGCATCTAGATCAACGTTACAC
CsFAE1_B 1198 CTTGAGAAGAGCTTAGGACTATCGCCAATCGATGTGGAGGCATCTAGATCAACGTTACAT
CsFAE1_C 1198 CTTGAGAAGAGCTTAGGACTATCGCCAATCGATGTGGAGGCATCTAGATCAACGTTACAT
At_FAE1  1201 CTAGAGAAGAACTTAGGACTATCGCCGATCGATGTGGAGGCATCTAGATCAACGTTACAT

                        N
CsFAE1_A 1258 AGATTTGGGAATACTTCGTCTAGCTCAATTTGGTATGAATTGGCATACATAGAAGCAAAA
CsFAE1_B 1258 AGATTTGGGAATACTTCGTCTAGCTCAATTTGGTATGAATTGGCATACATAGAAGCAAAA
CsFAE1_C 1258 AGATTTGGGAATACTTCGTCTAGCTCAATTTGGTATGAATTGGCATACATAGAAGCAAAA
At_FAE1  1261 AGATTTGGGAATACTTCATCTAGCTCAATTTGGTATGAATTAGCATACATAGAGGCAAAG

                                                                        C
CsFAE1_A 1318 GGAAGGATGAAGAAAGGGAATAGAGCTTGGCAGATTGCTTTAGGGTCAGGATTTAAGTGT
CsFAE1_B 1318 GGAAGGATGAAGAAAGGGAATAGAGCTTGGCAGATTGCTTTAGGGTCAGGGTTTAAGTGT
CsFAE1_C 1318 GGAAGGATGAAGAAAGGGAATAGAGCTTGGCAGATTGCTTTAGGGTCAGGGTTTAAGTGT
At_FAE1  1321 GGAAGAATGAAGAAAGGGAATAAAGCTTGGCAGATTGCTTTAGGATCAGGGTTTAAGTGT

CsFAE1_A 1378 AACAGTGCGGTTTGGGTGGCTCTATGCAATGTCAAGGCTTCGGCGAATAGTCCTTGGGAA
CsFAE1_B 1378 AACAGTGCGGTTTGGGTGGCTCTATGCAATGTCAAGGCTTCGGCGAATAGTCCTTGGGAA
CsFAE1_C 1378 AACAGTGCGGTTTGGGTGGCTCTATGCAATGTCAAGGCTTCCGCGAATAGTCCTTGGGAA
At_FAE1  1381 AATAGTGCGGTTTGGGTGGCTCTACGCAATGTCAAGGCATCGGCAAATAGTCCTTGGCAA
```

```
CsFAE1_A 1438 CATTGCATCGATAGATATCCGGTTCAAATTGATTCTGGTTCATCAAAATCAGATACTCAT
CsFAE1_B 1438 GATTGCATCGATAGATATCCGGTTCAAATTGATTCTGATTCATCAAAATCAGAGACTCAT
CsFAE1_C 1438 CATTGCATCGATAGATATCCGGTTCAAATTGATTCTGATTCATCAAAATCAGAGACTCAT
At_FAE1  1441 CATTGCATCGATAGATATCCGGTTAAAATTGATTCTGATTTGTCAAAGTCAAAGACTCAT

CsFAE1_A 1498 GTCAAAAACGGTCGGTCCTAA
CsFAE1_B 1498 GTCAAAAACGGTCGGTCCTAA
CsFAE1_C 1498 GTCAAAAACGGTCGGTCCTAA
At_FAE1  1501 GTCCAAAACGGTCGGTCCTAA
```
