# Supplementary material for: Polyploid genome of Camelina sativa revealed by isolation of fatty acid synthesis genes
Source: BMC Plant Biol. 2010 Oct 27;10:233. doi: 10.1186/1471-2229-10-233 (PMC3017853; doi:10.1186/1471-2229-10-233)
Supplement: Additional file 3 — Camelineae FAD2 and FAE1 protein alignment. (A) Amino acid sequence comparison of FAD2 sequences from species in the tribe Camelineae. The amino acid at position 44 is indicated with a blue underline while the green underline indicates the ER localization signal [45]. The three His boxes described by Tocher et al [44] are indicated with red boxes. The Arabidopsis thaliana FAD2 sequence was obtained from Genbank [Genbank:NP_187819]. (B) Amino acid sequence comparison of FAE1 sequences from species in the tribe Camelineae. Blue underlines below the sequence indicate the asparagine at amino acid position 424 and the highly conserved histidine and cysteine residues described by Ghanevati and Jaworski [49,50]. The red box indicates the region highly conserved among condensing enzymes in very long chain fatty acid biosynthesis [62]. The Arabidopsis thaliana FAE1 sequence was obtained from Genbank [Genbank:NP_195178]. [file 1471-2229-10-233-S3.PDF]

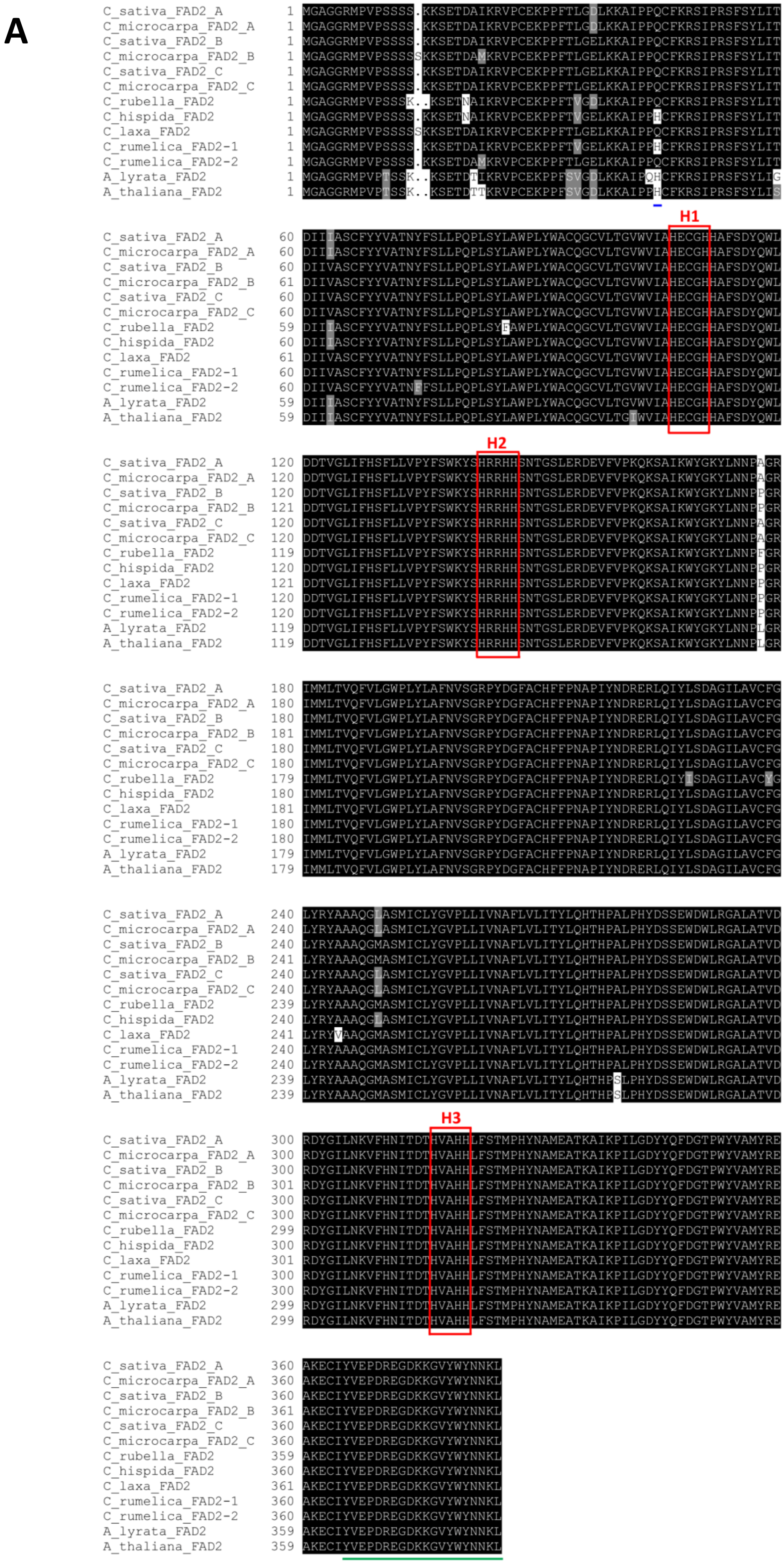
A
H1
H2
H3
C_sativa_FAD2_A
C_microcarpa_FAD2_A
C_sativa_FAD2_B
C_microcarpa_FAD2_B
C_sativa_FAD2_C
C_microcarpa_FAD2_C
C_rubella_FAD2
C_hispida_FAD2
C_laxa_FAD2
C_rumelica_FAD2-1
C_rumelica_FAD2-2
A_lyrata_FAD2
A_thaliana_FAD2

B

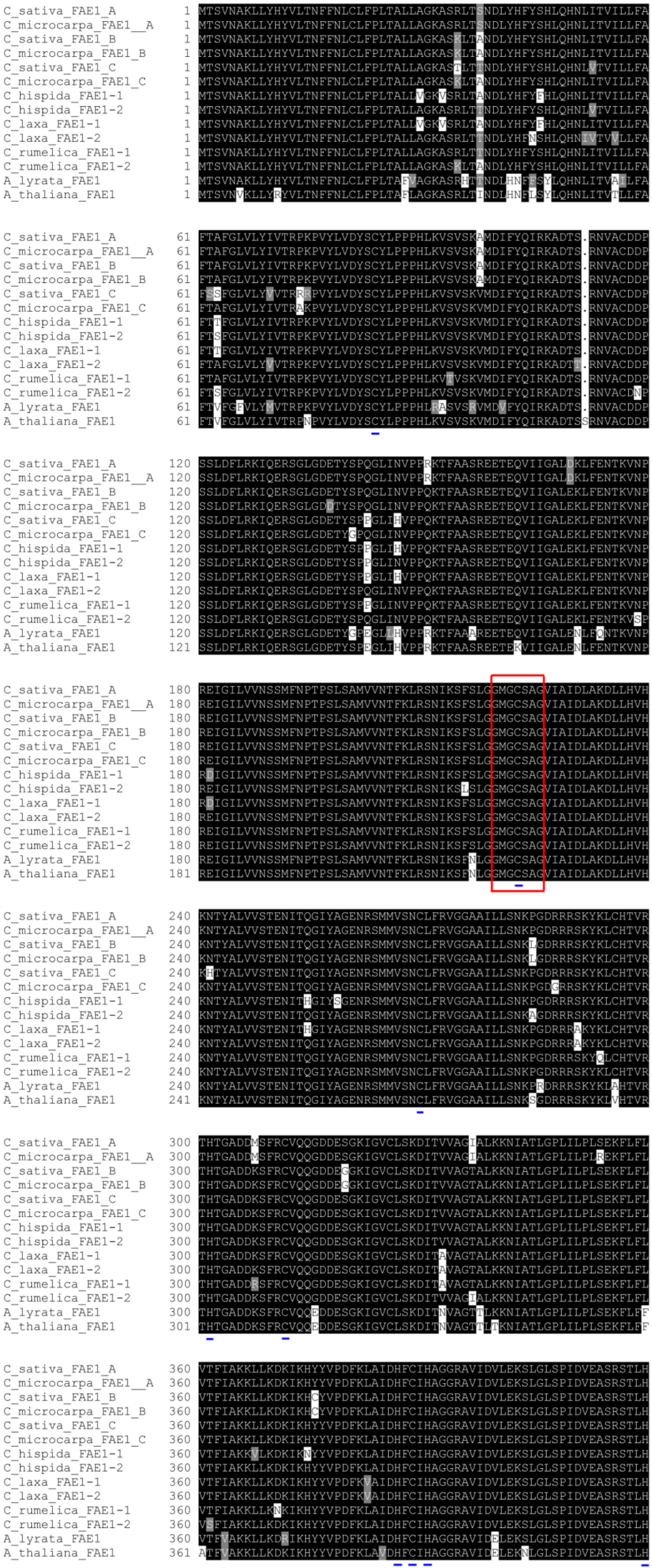
C_sativa_FAE1_A 1 MTSVNAKLLYHYVLTNFFNLCLFPLTALLAGKASRLTSNDLYHFYSHLQHNLITVILLFA
C_microcarpa_FAE1__A 1 MTSVNAKLLYHYVLTNFFNLCLFPLTALLAGKASRLTSNDLYHFYSHLQHNLITVILLFA
C_sativa_FAE1_B 1 MTSVNAKLLYHYVLTNFFNLCLFPLTALLAGKASKLTANDLYHFYSHLQHNLITVILLFA
C_microcarpa_FAE1_B 1 MTSVNAKLLYHYVLTNFFNLCLFPLTALLAGKASKLTANDLYHFYSHLQHNLITVILLFA
C_sativa_FAE1_C 1 MTSVNAKLLYHYVLTNFFNLCLFPLTALLAGKASTLTTNDLYHFYSHLQHNLVTVILLFA
C_microcarpa_FAE1_C 1 MTSVNAKLLYHYVLTNFFNLCLFPLTALLAGKASKLTANDLYHFYSHLQHNLITVILLFA
C_hispida_FAE1-1 1 MTSVNAKLLYHYVLTNFFNLCLFPLTALLVGKVSRLTANDLYHFYFHLQHNLITVILLFA
C_hispida_FAE1-2 1 MTSVNAKLLYHYVLTNFFNLCLFPLTALLAGKASRLTTNDLYHFYSHLQHNLVTVILLFA
C_laxa_FAE1-1 1 MTSVNAKLLYHYVLTNFFNLCLFPLTALLVGKVSRLTANDLYHFYFHLQHNLITVILLFA
C_laxa_FAE1-2 1 MTSVNAKLLYHYVLTNFFNLCLFPLTALLAGKASRLTTNDLYHFNSHLQHNIVTVVLLFA
C_rumelica_FAE1-1 1 MTSVNAKLLYHYVLTNFFNLCLFPLTALLAGKASRLTTNDLYHFYSHLQHNLITVILLFA
C_rumelica_FAE1-2 1 MTSVNAKLLYHYVLTNFFNLCLFPLTALLAGKASKLTANDLYHFYSHLQHNLITVILLFA
A_lyrata_FAE1 1 MTSVNAKLLYHYVLTNFFNLCLFPLTAFVAGKASRHTTNDLHNFFSYLQHNLITVAILFA
A_thaliana_FAE1 1 MTSVNVKLLYRYVLTNFFNLCLFPLTAFLAGKASRLTINDLHNFLSYLQHNLITVTLLFA
C_sativa_FAE1_A 61 FTAFGLVLYIVTRPKPVYLVDYSCYLPPPHLKVSVSKAMDIFYQIRKADTS.RNVACDDP
C_microcarpa_FAE1__A 61 FTAFGLVLYIVTRPKPVYLVDYSCYLPPPHLKVSVSKAMDIFYQIRKADTS.RNVACDDP
C_sativa_FAE1_B 61 FTAFGLVLYIVTRPKPVYLVDYSCYLPPPHLKVSVSKAMDIFYQIRKADTS.RNVACDDP
C_microcarpa_FAE1_B 61 FTAFGLVLYIVTRPKPVYLVDYSCYLPPPHLKVSVSKAMDIFYQIRKADTS.RNVACDDP
C_sativa_FAE1_C 61 FSSFGLVLYVVTRRRPVYLVDYSCYLPPPHLKVSVSKVMDIFYQIRKADTS.RNVACDDP
C_microcarpa_FAE1_C 61 FTAFGLVLYIVTRAKPVYLVDYSCYLPPPHLKVSVSKVMDIFYQIRKADTS.RNVACDDP
C_hispida_FAE1-1 61 FTTFGLVLYIVTRPKPVYLVDYSCYLPPPHLKVSVSKVMDIFYQIRKADTS.RNVACDDP
C_hispida_FAE1-2 61 FTSFGLVLYIVTRPKPVYLVDYSCYLPPPHLKVSVSKVMDIFYQIRKADTS.RNVACDDP
C_laxa_FAE1-1 61 FTTFGLVLYIVTRPKPVYLVDYSCYLPPPHLKVSVSKVMDIFYQIRKADTS.RNVACDDP
C_laxa_FAE1-2 61 FTAFGLVLYVVTRPKPVYLVDYSCYLPPPHLKVSVSKVMDIFYQIRKADTT.RNVACDDP
C_rumelica_FAE1-1 61 FTAFGLVLYIVTRPKPVYLVDYSCYLPPPHLKVTVSKVMDIFYQIRKADTS.RNVACDDP
C_rumelica_FAE1-2 61 FTSFGLVLYIVTRPKPVYLVDYSCYLPPPHLKVSVSKVMDIFYQIRKADTS.RNVACDNP
A_lyrata_FAE1 61 FTVFGFVLYMVTRPKPVYLVDYSCYLPPPHLRASVSRVMDVFYQIRKADTS.RNVACDDP
A_thaliana_FAE1 61 FTVFGLVLYIVTRPNPVYLVDYSCYLPPPHLKVSVSKVMDIFYQIRKADTSSRNVACDDP
C_sativa_FAE1_A 120 SSLDFLRKIQERSGLGDETYSPQGLINVPPRKTFAASREETEQVIIGALDKLFENTKVNP
C_microcarpa_FAE1__A 120 SSLDFLRKIQERSGLGDETYSPQGLINVPPRKTFAASREETEQVIIGALDKLFENTKVNP
C_sativa_FAE1_B 120 SSLDFLRKIQERSGLGDETYSPQGLINVPPQKTFAASREETEQVIIGALEKLFENTKVNP
C_microcarpa_FAE1_B 120 SSLDFLRKIQERSGLGDDTYSPQGLINVPPQKTFAASREETEQVIIGALEKLFENTKVNP
C_sativa_FAE1_C 120 SSLDFLRKIQERSGLGDETYSPPGLIHVPPQKTFAASREETEQVIIGALEKLFENTKVNP
C_microcarpa_FAE1_C 120 SSLDFLRKIQERSGLGDETYGPQGLINVPPQKTFAASREETEQVIIGALEKLFENTKVNP
C_hispida_FAE1-1 120 SSLDFLRKIQERSGLGDETYSPPGLIHVPPQKTFAASREETEQVIIGALEKLFENTKVNP
C_hispida_FAE1-2 120 SSLDFLRKIQERSGLGDETYSPPGLINVPPQKTFAASREETEQVIIGALEKLFENTKVNP
C_laxa_FAE1-1 120 SSLDFLRKIQERSGLGDETYSPPGLIHVPPQKTFAASREETEQVIIGALEKLFENTKVNP
C_laxa_FAE1-2 120 SSLDFLRKIQERSGLGDETYSPQGLINVPPQKTFAASREETEQVIIGALEKLFENTKVNP
C_rumelica_FAE1-1 120 SSLDFLRKIQERSGLGDETYSPPGLINVPPQKTFAASREETEQVIIGALEKLFENTKVNP
C_rumelica_FAE1-2 120 SSLDFLRKIQERSGLGDETYSPQGLINVPPQKTFAASREETEQVIIGALEKLFENTKVSP
A_lyrata_FAE1 120 SSLDFLRKIQERSGLGDETYGPEGLLHVPPRKTFAAAREETEQVIIGALENLFQNTKVNP
A_thaliana_FAE1 121 SSLDFLRKIQERSGLGDETYSPEGLIHVPPRKTFAASREETEKVIIGALENLFENTKVNP
C_sativa_FAE1_A 180 REIGILVVNSSMFNPTPSLSAMVVNTFKLRSNIKSFSLGGMGCSAGVIAIDLAKDLLHVH
C_microcarpa_FAE1__A 180 REIGILVVNSSMFNPTPSLSAMVVNTFKLRSNIKSFSLGGMGCSAGVIAIDLAKDLLHVH
C_sativa_FAE1_B 180 REIGILVVNSSMFNPTPSLSAMVVNTFKLRSNIKSFSLGGMGCSAGVIAIDLAKDLLHVH
C_microcarpa_FAE1_B 180 REIGILVVNSSMFNPTPSLSAMVVNTFKLRSNIKSFSLGGMGCSAGVIAIDLAKDLLHVH
C_sativa_FAE1_C 180 REIGILVVNSSMFNPTPSLSAMVVNTFKLRSNIKSFSLGGMGCSAGVIAIDLAKDLLHVH
C_microcarpa_FAE1_C 180 REIGILVVNSSMFNPTPSLSAMVVNTFKLRSNIKSFSLGGMGCSAGVIAIDLAKDLLHVH
C_hispida_FAE1-1 180 RDIGILVVNSSMFNPTPSLSAMVVNTFKLRSNIKSFSLGGMGCSAGVIAIDLAKDLLHVH
C_hispida_FAE1-2 180 REIGILVVNSSMFNPTPSLSAMVVNTFKLRSNIKSLSLGGMGCSAGVIAIDLAKDLLHVH
C_laxa_FAE1-1 180 RDIGILVVNSSMFNPTPSLSAMVVNTFKLRSNIKSFSLGGMGCSAGVIAIDLAKDLLHVH
C_laxa_FAE1-2 180 REIGILVVNSSMFNPTPSLSAMVVNTFKLRSNIKSFSLGGMGCSAGVIAIDLAKDLLHVH
C_rumelica_FAE1-1 180 REIGILVVNSSMFNPTPSLSAMVVNTFKLRSNIKSFSLGGMGCSAGVIAIDLAKDLLHVH
C_rumelica_FAE1-2 180 REIGILVVNSSMFNPTPSLSAMVVNTFKLRSNIKSFSLGGMGCSAGVIAIDLAKDLLHVH
A_lyrata_FAE1 180 REIGILVVNSSMFNPTPSLSAMVVNTFKLRSNIKSFNLGGMGCSAGVIAIDLAKDLLHVH
A_thaliana_FAE1 181 REIGILVVNSSMFNPTPSLSAMVVNTFKLRSNIKSFNLGGMGCSAGVIAIDLAKDLLHVH
C_sativa_FAE1_A 240 KNTYALVVSTENITQGIYAGENRSMMVSNCLFRVGGAAILLSNKPGDRRRSKYKLCHTVR
C_microcarpa_FAE1__A 240 KNTYALVVSTENITQGIYAGENRSMMVSNCLFRVGGAAILLSNKPGDRRRSKYKLCHTVR
C_sativa_FAE1_B 240 KNTYALVVSTENITQGIYAGENRSMMVSNCLFRVGGAAILLSNKLGDRRRSKYKLCHTVR
C_microcarpa_FAE1_B 240 KNTYALVVSTENITQGIYAGENRSMMVSNCLFRVGGAAILLSNKLGDRRRSKYKLCHTVR
C_sativa_FAE1_C 240 KHTYALVVSTENITQGIYAGENRSMMVSNCLFRVGGAAILLSNKPGDRRRSKYKLCHTVR
C_microcarpa_FAE1_C 240 KNTYALVVSTENITQGIYAGENRSMMVSNCLFRVGGAAILLSNKPGDGRRSKYKLCHTVR
C_hispida_FAE1-1 240 KNTYALVVSTENITHGIYSGENRSMMVSNCLFRVGGAAILLSNKPGDRRRSKYKLCHTVR
C_hispida_FAE1-2 240 KNTYALVVSTENITQGIYAGENRSMMVSNCLFRVGGAAILLSNKAGDRRRSKYKLCHTVR
C_laxa_FAE1-1 240 KNTYALVVSTENITHGIYAGENRSMMVSNCLFRVGGAAILLSNKPGDRRAKYKLCHTVR
C_laxa_FAE1-2 240 KNTYALVVSTENITQGIYAGENRSMMVSNCLFRVGGAAILLSNKPGDRRAKYKLCHTVR
C_rumelica_FAE1-1 240 KNTYALVVSTENITQGIYAGENRSMMVSNCLFRVGGAAILLSNKPGDRRRSKYQLCHTVR
C_rumelica_FAE1-2 240 KNTYALVVSTENITQGIYAGENRSMMVSNCLFRVGGAAILLSNKPGDRRRSKYKLCHTVR
A_lyrata_FAE1 240 KNTYALVVSTENITQGIYAGENRSMMVSNCLFRVGGAAILLSNKPRDRRRSKYKLAHTVR
A_thaliana_FAE1 241 KNTYALVVSTENITQGIYAGENRSMMVSNCLFRVGGAAILLSNKSGDRRRSKYKLVHTVR
C_sativa_FAE1_A 300 THTGADDMSFRCVQQGDDESGKIGVCLSKDITVVAGIALKKNIATLGPLILPLSEKFLFL
C_microcarpa_FAE1__A 300 THTGADDMSFRCVQQGDDESGKIGVCLSKDITVVAGIALKKNIATLGPLILPLREKFLFL
C_sativa_FAE1_B 300 THTGADDKSFRCVQQGDDEGGKIGVCLSKDITVVAGTALKKNIATLGPLILPLSEKFLFL
C_microcarpa_FAE1_B 300 THTGADDKSFRCVQQGDDEGGKIGVCLSKDITVVAGTALKKNIATLGPLILPLSEKFLFL
C_sativa_FAE1_C 300 THTGADDKSFRCVQQGDDESGKIGVCLSKDITVVAGTALKKNIATLGPLILPLSEKFLFL
C_microcarpa_FAE1_C 300 THTGADDKSFRCVQQGDDESGKIGVCLSKDITVVAGTALKKNIATLGPLILPLSEKFLFL
C_hispida_FAE1-1 300 THTGADDKSFRCVQQGDDESGKIGVCLSKDITVVAGTALKKNIATLGPLILPLSEKFLFL
C_hispida_FAE1-2 300 THTGADDKSFRCVQQGDDESGKIGVCLSKDITVVAGTALKKNIATLGPLILPLSEKFLFL
C_laxa_FAE1-1 300 THTGADDKSFRCVQQGDDESGKIGVCLSKDITAVAGTALKKNIATLGPLILPLSEKFLFL
C_laxa_FAE1-2 300 THTGADDKSFRCVQQGDDESGKIGVCLSKDITAVAGTALKKNIATLGPLILPLSEKFLFL
C_rumelica_FAE1-1 300 THTGADDRSFRCVQQGDDESGKIGVCLSKDITAVAGTALKKNIATLGPLILPLSEKFLFL
C_rumelica_FAE1-2 300 THTGADDKSFRCVQQGDDESGKIGVCLSKDITVVAGIALKKNIATLGPLILPLSEKFLFL
A_lyrata_FAE1 300 THTGADDKSFRCVQQEDDESGKIGVCLSKDITNVAGTTLKKNIATLGPLILPLSEKFLFF
A_thaliana_FAE1 301 THTGADDKSFRCVQQEDDESGKIGVCLSKDITNVAGTTLTKNIATLGPLILPLSEKFLFF
C_sativa_FAE1_A 360 VTFIAKKLLKDKIKHYYVPDFKLAIDHFCIHAGGRAVIDVLEKSLGLSPIDVEASRSTLH
C_microcarpa_FAE1__A 360 VTFIAKKLLKDKIKHYYVPDFKLAIDHFCIHAGGRAVIDVLEKSLGLSPIDVEASRSTLH
C_sativa_FAE1_B 360 VTFIAKKLLKDKIKHCYVPDFKLAIDHFCIHAGGRAVIDVLEKSLGLSPIDVEASRSTLH
C_microcarpa_FAE1_B 360 VTFIAKKLLKDKIKHCYVPDFKLAIDHFCIHAGGRAVIDVLEKSLGLSPIDVEASRSTLH
C_sativa_FAE1_C 360 VTFIAKKLLKDKIKHYYVPDFKLAIDHFCIHAGGRAVIDVLEKSLGLSPIDVEASRSTLH
C_microcarpa_FAE1_C 360 VTFIAKKLLKDKIKHYYVPDFKLAIDHFCIHAGGRAVIDVLEKSLGLSPIDVEASRSTLH
C_hispida_FAE1-1 360 VTFIAKKVLKDKIKNYYVPDFKLAIDHFCIHAGGRAVIDVLEKSLGLSPIDVEASRSTLH
C_hispida_FAE1-2 360 VTFIAKKLLKDKIKHYYVPDFKLAIDHFCIHAGGRAVIDVLEKSLGLSPIDVEASRSTLH
C_laxa_FAE1-1 360 VTFIAKKLLKDKIKHYYVPDFKVAIDHFCIHAGGRAVIDVLEKSLGLSPIDVEASRSTLH
C_laxa_FAE1-2 360 VTFIAKKLLKDKIKHYYVPDFKVAIDHFCIHAGGRAVIDVLEKSLGLSPIDVEASRSTLH
C_rumelica_FAE1-1 360 VTFIAKKLLKNKIKHYYVPDFKLAIDHFCIHAGGRAVIDVLEKSLGLSPIDVEASRSTLH
C_rumelica_FAE1-2 360 VSFIAKKLLKDKIKHYYVPDFKLAIDHFCIHAGGRAVIDVLEKSLGLSPIDVEASRSTLH
A_lyrata_FAE1 360 VTFVAKKLLKDRIKHYYVPDFKLAIDHFCIHAGGRAVIDELEKSLGLSPIDVEASRSTLH
A_thaliana_FAE1 361 ATFVAKKLLKDKIKHYYVPDFKLAVDHFCIHAGGRAVIDELEKNLGLSPIDVEASRSTLH

```
C_sativa_FAE1_A        420 RFGNTSSSSIWYELAYIEAKGRMKKGNRAWQIALGSGFKCNSAVWVALCNVKASANSPWE
C_microcarpa_FAE1__A   420 RFGNTSSSSIWYELAYIEAKGRMKKGNRAWQIALGSGFKCNSAVWVALCNVKASANSPWE
C_sativa_FAE1_B        420 RFGNTSSSSIWYELAYIEAKGRMKKGNRAWQIALGSGFKCNSAVWVALCNVKASANSPWE
C_microcarpa_FAE1_B    420 RFGNTSSSSIWYELAYIEAKGRMKKGNRAWQIALGSGFKCNSAVWVALCNVKASANSPWE
C_sativa_FAE1_C        420 RFGNTSSSSIWYELAYIEAKGRMKKGNRAWQIALGSGFKCNSAVWVALCNVKASANSPWE
C_microcarpa_FAE1_C    420 RFGNTSSSSIWYELAYIEAKGRMKKGNRAWQIALGSGFKCNSAVWVALCNVKASANSPWE
C_hispida_FAE1-1       420 RFGNTSSSSIWYELAYIEAKGRMKKGNRAWQIALGSGFKCNSAVWVALCNVKASANSPWE
C_hispida_FAE1-2       420 RFGNTSSSSIWYELAYIEAKGRMKKGNRAWQIALGSGFKCNSAVWVALCNVKASANSPWE
C_laxa_FAE1-1          420 RFGNTSSSSIWYELAYIEAKGRMKKGNKAWQIALGSGFKCNSAVWVALCNVKASANSPWE
C_laxa_FAE1-2          420 RFGNTSSSSIWYELAYIEAKGRMKKGNKAWQIALGSGFKCNSAVWVALCNVKASANSPWE
C_rumelica_FAE1-1      420 RFGNTSSSSIWYELAYIEAKGRMKKGNRAWQIALGSGFKCNSAVWVALCNVKASANSPWE
C_rumelica_FAE1-2      420 RFGNTSSSSIWYELAYTEAKGRMKKGNRAWQIALGSGFKCNSAVWVALCNVKASANSPWE
A_lyrata_FAE1          420 RFGNTSSSSIWYELAYIEAKGRMKKGNKAWQIALGSGFKCNSAVWVALRNVKPSANSPWQ
A_thaliana_FAE1        421 RFGNTSSSSIWYELAYIEAKGRMKKGNKAWQIALGSGFKCNSAVWVALRNVKASANSPWQ

C_sativa_FAE1_A        480 HCIDRYPVQIDSGSSKSDTHVKNGRS
C_microcarpa_FAE1__A   480 HCIDRYPVQIDSGSSKSDTHVKNGRS
C_sativa_FAE1_B        480 DCIDRYPVQIDSDSSKSETHVKNGRS
C_microcarpa_FAE1_B    480 DCIDRYPVQIDSDSSKSETHVKNGRS
C_sativa_FAE1_C        480 HCIDRYPVQIDSDSSKSETHVKNGRS
C_microcarpa_FAE1_C    480 HCIDRYPVQIDSDSSKLETHVKNGRS
C_hispida_FAE1-1       480 HCIDRYPVQIDSDSSKSETHVKNGRS
C_hispida_FAE1-2       480 HCIDRYPVQIDSDSSKSETHVKNGRS
C_laxa_FAE1-1          480 HCIDRYPVQIDFDSSKSDTHVKNGRS
C_laxa_FAE1-2          480 HCIDRYPVQIDFDSSKSDTHVKNGRS
C_rumelica_FAE1-1      480 HCIDRYPVQLNSDSSKSETHVKNGRS
C_rumelica_FAE1-2      480 HCIDRYPVKIDSDSSKSETHVKNGRS
A_lyrata_FAE1          480 HCIDRYPAKIDSDLSKSETHVKNGRS
A_thaliana_FAE1        481 HCIDRYPVKIDSDLSKSKTHVQNGRS
```
