# Supplementary material for: Polyploid genome of Camelina sativa revealed by isolation of fatty acid synthesis genes
Source: BMC Plant Biol. 2010 Oct 27;10:233. doi: 10.1186/1471-2229-10-233 (PMC3017853; doi:10.1186/1471-2229-10-233)
Supplement: Additional file 4 — Primers used for qPCR analyses. List of primers used for qPCR analyses [file 1471-2229-10-233-S4.DOCX]

## Additional File 4 - Primers used for qPCR analyses

|  | **Primer or Probe Name** | **Sequence (5’ – 3’)** |
| --- | --- | --- |
| qPCR of CsACTIN | CsACT For | ACA ATT TCC CGC TCT GCT GTT GTG |
|  | CsACT Rev | AGG GTT TCT CTC TTC CAC ATG CCA |
|  | CsACT probe | FAM - TGT TTC AAA CGC TCT ATC CCT CGC TC – IABLFQ |
| qPCR of CsFAD2 | CsFAD2 A For1 | CTG CGA GAA ACC ACC GTT CAC CC |
|  | CsFAD2 all Rev | CAC GAG TAG TCA ACG AGG TAA ACC GG |
|  | CsFAD2 all probe | FAM - CCA CTT CTA TTC CCA TCT CCA ACA CAA CC - IABLFQ |
| qPCR of CsFAE1 | CsFAE1 all For | AAC CTT TGC TTG TTT CCG TTA ACG GC |
|  | CsFAE1 all Rev | CAC GAG TAG TCA ACG AGG TAA ACC GG |
|  | CsFAE1 all probe | FAM - CCA CTT CTA TTC CCA TCT CCA ACA CAA CC - IABLFQ |
