# Supplementary material for: Polyploid genome of Camelina sativa revealed by isolation of fatty acid synthesis genes
Source: BMC Plant Biol. 2010 Oct 27;10:233. doi: 10.1186/1471-2229-10-233 (PMC3017853; doi:10.1186/1471-2229-10-233)
Supplement: Additional file 5 — SNPs distinguishing each copy of CsFAD2 and CsFAE1. List of SNPs used in Sequenom MassARRAY™ analyses to distinguish the three copies of CsFAD2 and of CsFAE1 [file 1471-2229-10-233-S5.DOCX]

**Additional File 5 – SNPs distinguishing each copy of *CsFAD2* and *CsFAE1***

| **SNP_ID** | **Nucleotide position from beginning of coding region** |
| --- | --- |
| FAD2_A4 | 51 |
| FAD2_A2 | 453 |
| FAD2_A6 | 549 |
| FAD2_B4 | 288 |
| FAD2_B5 | 687 |
| FAD2_B8 | 1109 |
| FAD2_C1 | 78 |
| FAD2_C5 | 615 |
| FAD2_C3 | 966 |
| FAE1_A4 | 624 |
| FAE1_A3 | 1368 |
| FAE1_A7 | 1475 |
| FAE1_B4 | 414 |
| FAE1_B5 | 783 |
| FAE1_B8 | 1438 |
| FAE1_C1 | 336 |
| FAE1_C2 | 721 |
| FAE1_C7 | 1419 |
| FAE1_ABC1 | 104 |
