# Supplementary figures and images for: Polyploid genome of Camelina sativa revealed by isolation of fatty acid synthesis genes
Source: BMC Plant Biol. 2010 Oct 27;10:233. doi: 10.1186/1471-2229-10-233 (PMC3017853; doi:10.1186/1471-2229-10-233)

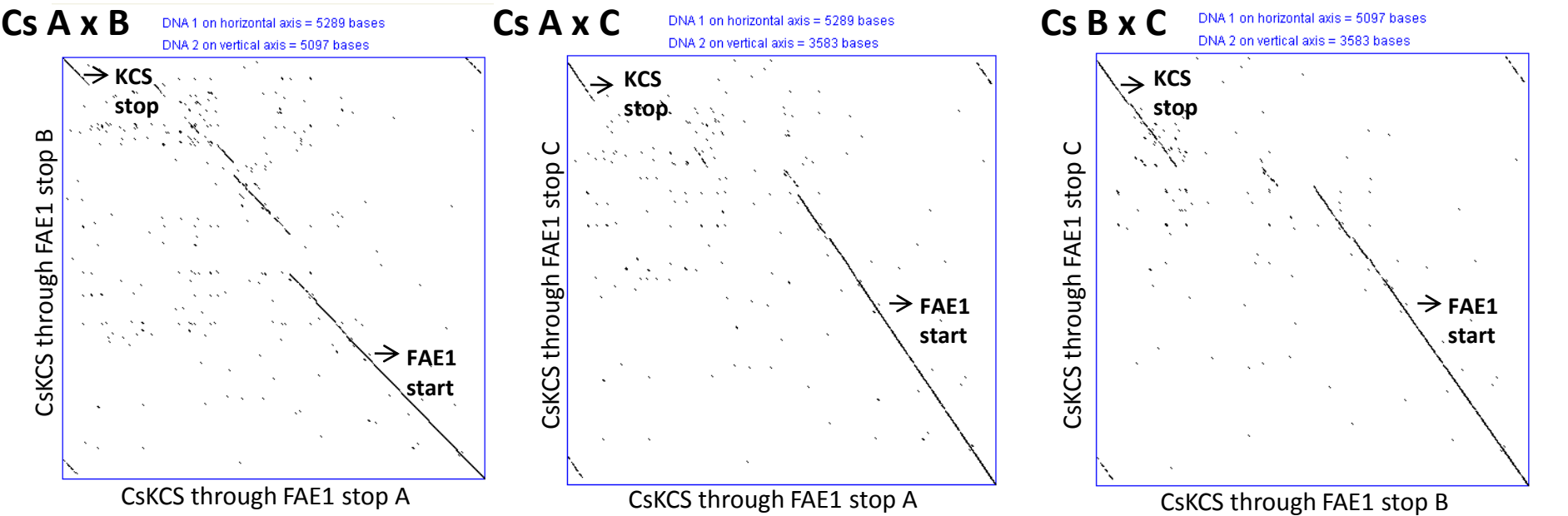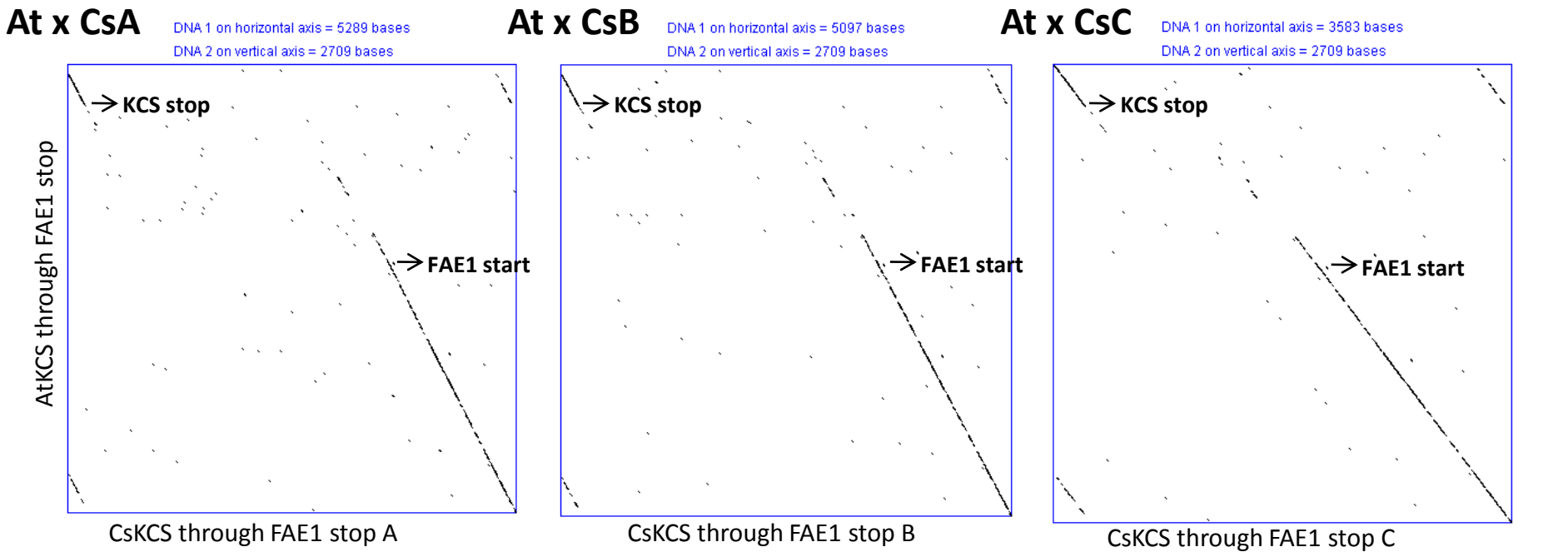

Supplement: Additional file 6 — Dot plots of KCS17-FAE1 intergenic region. Sequences obtained for CsKCS17-FAE1A, B and C were aligned with each other and with Arabidopsis orthologous region two at a time in a dot plot with parameters set for perfect conservation on a sliding window of 9 bases. [file 1471-2229-10-233-S6.PDF]
