# Supplementary material for: Polyploid genome of Camelina sativa revealed by isolation of fatty acid synthesis genes
Source: BMC Plant Biol. 2010 Oct 27;10:233. doi: 10.1186/1471-2229-10-233 (PMC3017853; doi:10.1186/1471-2229-10-233)
Supplement: Additional file 7 — Deep sequencing reads for 6 predicted single-copy genes in C. sativa. Sequences determined by 454 sequencing of cDNA from 15 DPA C. sativa seeds, aligned with 6 genes predicted by Duarte et al [35] to be single-copy in flowering plants. [file 1471-2229-10-233-S7.PDF]

```
Query= AT4G37830.1 | Symbols:  | cytochrome c oxidase-related |chr4:17787473-17788828 REVERSE
(574 letters)
hits: 80
haplotypes: 3

804_0          131 agacatccgtcgctcctaagcgaaacttttcctcttccgccggccatgacgatgcttatg 190
FA2ZECP01E1P5A 33  ...................a....g...c..............................t 92
FA2ZECP01BL8NN 33  ...................a....g...c..............................t 92
FA2ZECP01EO1E9 33  ...................a....g...c..............................t 92
FA2ZECP02JP58J 41    .................a....g...c............................... 98
FA2ZECP01C2P0P 117 ...................a....g...c............................... 176
FA2ZECP01DGJBS 117 ...................a....g...c............................... 176
FA2ZECP01EEHP4 117 ...................a....g...c............................... 176
FA2ZECP01DPZ21 117 ...................a....g...c............................... 176
FA2ZECP01DCG4R 110 ...................a....g...c............................... 169
FA2ZECP01BE5Z6 117 ...................a....g...c............................... 176
FA2ZECP01A5Y4Y 116 ...................a....g...c............................... 175
FA2ZECP02GM89K 123 ...................a....g...c............................... 182
FA2ZECP01C1GNO 117 ...................a....g...c............................... 176
FA2ZECP01A4C68 128 .a.................a....g...c............................... 187
FA2ZECP01A4JIQ 128 .a.................a....g...c............................... 187
FA2ZECP02IPIS1 101 .a.................a....g...c............................... 160
FA2ZECP02GQ6TJ 101 .a.................a....g...c............................... 160
FA2ZECP02GB1FW 101 .a.................a....g...c............................... 160
FA2ZECP01D55B9 105 .a.................a....g...c............................... 164
FA2ZECP01DHJJR 110 .a.................a....g...c............................... 169
FA2ZECP01A1B4R 110 .a.................a....g...c............................... 169
FA2ZECP01A6V0U 110 .a.................a....g...c............................... 169
FA2ZECP02GDG9O 120 .a.................a....g...c............................... 179
FA2ZECP02GC48K 125 .a.................a....g...c............................... 184
FA2ZECP02IUW9J 124 .a.................a....g...c............................... 183
FA2ZECP01C2DGV 218 .....a.............a....g...c............................... 159
FA2ZECP01EY9OS 218 .....a.............a....g...c............................... 159
FA2ZECP02H2QAD 109 .....a.............a....g...c............................... 168
FA2ZECP01CXA84 109 .....a.............a....g...c............................... 168
FA2ZECP01BBWCS 109 .....a.............a....g...c............................... 168
FA2ZECP02IHU9F 111 .....a.............a....g...c............................... 170
FA2ZECP02FONEF 114 .....a.............a....g...c............................... 173
FA2ZECP01EQEB6 114 .....a.............a....g...c............................... 173
FA2ZECP01AJVJK 122 .....a.............a....g...c............................... 181
FA2ZECP01D114Y 126 .....a.............a....g...c............................... 185
FA2ZECP02HT7RA 144 .....a.............a....g...c............................... 203
FA2ZECP02I2I6O 144 .....a.............a....g...c............................... 203
FA2ZECP01CYJAG 101 .....a.............a.t..g...c............................... 160
FA2ZECP01BEM3H 144 .....a.............a....g...c............................... 203
FA2ZECP02IUEAU 126 .....a.............a....g...c............................... 185

55_0           420  cgctcgtctcaaaacctggagtcatggcggaaagacattaacgaagctgcgattctctag 479
FA2ZECP02JNZAD 160  ......c..............c....................a.....a..g.....a.. 101
FA2ZECP02JYWH3 163  ......c..............c....................a.....a..g.....a.. 104
FA2ZECP02F2NSC 155  ......c..............c....................a.....a..g.....a.. 96
FA2ZECP02FIEIL 183  ......c..............c....................a.....a..g.....a.. 124
FA2ZECP02JO7HN 120  ......c..............c....................a.....a..g.....a.. 61
FA2ZECP02H5YY4 121  ......c..............c....................a.....a..g.....a.. 62
FA2ZECP02JW3QN 159  ......c..............c....................a.....a..g.....a.. 218
FA2ZECP01CWKKH 159  ......c..............c....................a.....a..g.....a.. 218
FA2ZECP01DLERT 203  ......c..............c....................a........c.....a.. 144
FA2ZECP01CNVVX 164  t-....c..............c....................a........c.....a.. 106
FA2ZECP02JUYAN 137  ......c..............c....................a........c.....a.. 78
FA2ZECP02JMQLN 221  ......c..............c....................a........c.....a.. 162
FA2ZECP01CZM66 135  ......c..............c....................a..t.....c.....a.. 76
FA2ZECP02FMTBO 20   ......c..............c....................a..t.....c.....a.. 79

Query= AT1G65270.1 | Symbols:  | similar to hypothetical protein [Vitis vinifera] (GB:CAN71035.1)
| chr1:24245386-24248654 FORWARD (1240 letters)
hits: 60
haplotypes: 3
5_0          420  cgctcgtctcaaaacctggagtcatggcggaaagacattaacgaagctgcgattctctag 479
FA2ZECP02JNZAD 160  ......c..............c....................a.....a..g.....a.. 101
FA2ZECP02JYWH3 163  ......c..............c....................a.....a..g.....a.. 104
FA2ZECP02F2NSC 155  ......c..............c....................a.....a..g.....a.. 96
```

```
FA2ZECP02FIEIL 183  ......c..............c....................a.....a..g.....a.. 124
FA2ZECP02H5YY4 121  ......c..............c....................a.....a..g.....a.. 62
FA2ZECP02JW3QN 159  ......c..............c....................a.....a..g.....a.. 218
FA2ZECP01CWKKH 159  ......c..............c....................a.....a..g.....a.. 218
FA2ZECP02JO7HN 120  ......c..............c....................a.....a..g.....a.. 61
FA2ZECP01DZYT2 11                           ..................a.....a..g.....a.. 48
FA2ZECP01DLERT 203  ......c..............c....................a........c.....a.. 144
FA2ZECP01CNVVX 164  t-....c..............c....................a........c.....a.. 106
FA2ZECP02JUYAN 137  ......c..............c....................a........c.....a.. 78
FA2ZECP02JMQLN 221  ......c..............c....................a........c.....a.. 162
FA2ZECP02FMTBO 20   ......c..............c....................a..t.....c.....a.. 79
FA2ZECP01CZM66 135  ......c..............c....................a..t.....c.....a.. 76

Query= AT1G31600.1 | Symbols:  | oxidoreductase, 2OG-Fe(II) oxygenase family protein |
chr1:11312942-11316454 REVERSE (2223 letters)
hits: 60
haplotypes: 3
471_0          75  ccagctgagcttgagaagagaaagcacaagctcaaacgtcttgttcagtctcccaattcc 134
FA2ZECP02I6MR6 95  ....................g..............g.................a..c... 154
FA2ZECP02IEAJ9 110 ....................g..............g.................a..c... 169
FA2ZECP02F6ELM 105 ....................g..............g.................a..c... 164
FA2ZECP02J1BN3 99  ....................g..............g.................a..c... 158
FA2ZECP02IV0XP 63  ....................g..............g.................a..c... 122
FA2ZECP02ITNZW 105 ....................g..............g.................a..c... 164
FA2ZECP02HG4J3 95  ....................g..............g.................a..c... 154
FA2ZECP01AUUHE 176 ....................g..............g.................a..c... 117
FA2ZECP01BT1RV 106 ....................g..............g...............-....c... 164
FA2ZECP01A7RHT 95  ....................g..............g.................a..c... 154
FA2ZECP01AIHOF 74  ....................g..............g.................a..c... 133
FA2ZECP01EQ20U 67  ....................g..............g.................a..c... 126
FA2ZECP01BP44F 106 ....................g..............g.................a..c... 165
FA2ZECP02IUES4 58  ....................g..............g........c........a..c... 117
FA2ZECP02G3PIL 58  ....................g..............g........c........a..c... 117
FA2ZECP02I1WJ4 58  ....................g..............g........c........a..c... 117
FA2ZECP02GNZER 101 ....................g..............g........c........a..c... 160
FA2ZECP02IGDBB 114 ....................g..............g........c........a..c... 173
FA2ZECP02II91U 93  ....................g..............g........c........a..c... 152
FA2ZECP02GTLOW 89  ....................g..............g........c........a..c... 148
FA2ZECP02HCG0R 91  ....................g..............g........c........a..c... 150
FA2ZECP02GXLW6 76  ....................g..............g........c........a..c... 135
FA2ZECP02H5IQC 89  ....................g..............g........c........a..c... 148
FA2ZECP02JR6G9 89  ....................g..............g........c........a..c... 148
FA2ZECP02FL23C 69  ....................g..............g........c........a..c... 128
FA2ZECP02I3TID 62  ....................g..............g........c........a..c... 121
FA2ZECP02FITVJ 93  ....................g..............g........c........a..c... 152
FA2ZECP02JJOW0 90  ....................g..............g........c........a..c... 149
FA2ZECP02JDUTW 69  ....................g..............g........c........a..c... 128
FA2ZECP02JXEZ5 96  ....................g..............g........c........a..c... 155
FA2ZECP02H6G88 99  ....................g..............g........c........a..c... 158
FA2ZECP02F8EXG 62  ....................g..............g........c........a..c... 121
FA2ZECP01CFDIS 102 ....................g..............g........c........a..c... 161
FA2ZECP01DDTS2 91  ....................g..............g........c........a..c... 150
FA2ZECP01A7ZKL 99  ....................g..............g........c........a..c... 158
FA2ZECP01DN9BJ 101 ....................g..............g........c........a..c... 160
FA2ZECP01BJAV8 179 ....................g..............g........c........a..c... 120
FA2ZECP01DMQY0 101 ....................g..............g........c........a..c... 160
FA2ZECP01EFMIC 109 ....................g..............g........c........a..c... 168
FA2ZECP01D2UTV 88  ....................g..............g........c........a..c... 147
FA2ZECP01DG7HA 71  ....................g..............g........c........a..c... 130
FA2ZECP02HM9Q8 127 ....................g..............g.....c..c........a..c... 186
FA2ZECP02JVZ5C 114 ....................g..............g.....c..c........a..c... 173
FA2ZECP02H903R 92  ....................g..............g.....c..c........a..c... 151
FA2ZECP01A841P 93  ....................g..............g.....c..c........a..c... 152
FA2ZECP01C4EGS 109 ....................g..............g.....c..c........a..c... 168
FA2ZECP01DNZWP 212 ....................g..............g.....c..c........a..c... 153
FA2ZECP01B8PPH 93  ....................g..............g.....c..c........a..c... 152
FA2ZECP01DTP4S 109 ....................g..............g.....c..c........a..c... 168
FA2ZECP01CZ6O1 85  ....................g..............g.....c..c........a..c... 144
FA2ZECP01B14Q3 96  ....................g..............g.....c..c........a..c... 155
FA2ZECP01EJG2U 94  ....................g..............g.....c..c........a..c... 153
FA2ZECP01D911Q 86  ....................g..............g.....c..c........a..c... 145
```

```
FA2ZECP01D1PQZ 96  ....................g..............g.....c..c........a..c... 155
FA2ZECP02GU0MM 96  ....................g..............g.....c..c........        148
FA2ZECP02HGPR4 13              ........g..............g..............a........a 60 ?
FA2ZECP02J1V54 13              ........g..............g..............a........a 60 ?
FA2ZECP02JKMVE 13              ........g..............g..............a........a 60 ?
FA2ZECP02FX91O 13              ........g..............g..............a........a 60 ?
```

Query= AT5G08170.1 | Symbols: EMB1873, ATAIH | ATAIH/EMB1873 (AGMATINE IMINOHYDROLASE); agmatine deiminase | chr5:2628326-2631099 REVERSE (1541 letters)
hits: 60
haplotypes: 3

```
558_0          67   gaatcgccggcggaacacggctactacatgccggcggaatgggattctcatgctcaaact 126
FA2ZECP02F3OD9 23   .....a...........t....tt....................ac.............. 82
FA2ZECP01AJ789 49   .....a...........t....tt....................ac.............. 108
FA2ZECP02ID6TF 52   .....a...........t....tt.....n..............ac.............. 111
FA2ZECP01C551S 84   .....a...........t....tt....................ac.............. 143
FA2ZECP01EMW0W 83   .....a...........t....tt....................ac.............. 142
FA2ZECP01AYULY 84   .....a...........t....tt....................ac.............. 143
FA2ZECP01BO2WP 43   .....a........g.......tt....................ac.............. 102
FA2ZECP01BCKIE 53   .....a........g.......tt....................ac.............. 112
FA2ZECP01CN97A 49   .....a........g.......tt....................ac.............. 108
FA2ZECP02IIFK4 13         ........g.......tt....................ac.............. 66
FA2ZECP02FKN9K 47   .....a................tt....................ac.............. 106
FA2ZECP02JC1FW 16   .....a................tt....................ac.............. 75


558_0          187  gtgtttgcagatgttgcaaaggccatctcaaagttcgagcctgtcactgtctgtgctagc 246
FA2ZECP02F3OD9 143  ............a....t.............................a........a... 202
FA2ZECP01AJ789 169  ............a....t.............................a........a... 228
FA2ZECP02ID6TF 172  ............a....t.............................a........a... 231
FA2ZECP02FSIYT 233   ................t.............................a........a... 175
FA2ZECP01BO2WP 163  .................t.............................a........a... 222
FA2ZECP01BCKIE 173  .................t.............................a........a... 232
FA2ZECP02IIFK4 127  .................t.............................a........a... 186
FA2ZECP02FKN9K 167  ..a....t.......................................a........a..t 226
FA2ZECP02JC1FW 136  ..a....t.......................................a........a..t 195
```

Query= AT5G08060.1 | Symbols: | similar to unknown [Populus trichocarpa] (GB:ABK93975.1) | chr5:2580450-2582565 FORWARD (957 letters)
hits: 60
haplotypes: 3

```
882_0          310 aatcgtgccttcctcggatccagagaccgtatacaacatcgtataccacggcagagatca 369
FA2ZECP01DEIP0 130 c.........a..........c..a...........................c....... 189
FA2ZECP01E5HXX 185 c.........a..........c..a...........................c....... 244
FA2ZECP01BXALP 83  c.........a..........c..a...........................c....... 142
FA2ZECP01BRIFR 79  c.........a..........c..a...........................c....... 138
FA2ZECP02IVNXK 83  c.........a..........c..a...........................c....... 142
FA2ZECP02FQQDK 151 c.........a..........c..a..t........................c....... 92
FA2ZECP02HNNE4 83  c.........a..........c..a..t........................c....... 142
FA2ZECP02GYJZO 194 c...t.....a..........c..a..t........................c....... 135
FA2ZECP02F6Y18 192 c.........a..........c..a..t........................c......  250
FA2ZECP02GO010 151 c.........a..........c..a..t........................c....... 210
FA2ZECP02ICUYJ 206 c.........a..........c..a..t.........                        242
FA2ZECP02G6OM5 187 c.........a..........c..a..t........................c....... 246
FA2ZECP01AWVH8 206 c.........a..........c..a..t.........                        242
FA2ZECP01EGLDA 193 c.........a..........c..a..t........................c....    249
FA2ZECP02JYKYH 137 c........ga.a........c..a...........................c....... 78
FA2ZECP01BYQCJ 149 c........ga.a........c..a...........................c....... 90
FA2ZECP02JD0BI 139 c........ga.a........c..a...........................c....... 198
FA2ZECP02G655Q 205 c........ga.a........c..a.................                   246
FA2ZECP01EO78V 203 c........ga.a........c..a.................                   244
FA2ZECP02GIIEJ 207 c........ga.a........c..a................                    247
FA2ZECP02GTWUL 54  t........ga.a........c..a...................                 11
FA2ZECP01BIG2Y 208 c........ga.a........c..-.a..............                   247
FA2ZECP02G1EST 120 c........ga.a........c..a...........................c....... 179
```

Query= AT2G18040.1 | Symbols: PIN1AT | PIN1AT (parvulin 1At) | chr2:7849246-7850952 FORWARD (875 letters)
hits: 60

```
haplotypes: 3
915_0          71  aacgaagatagattcagatcggaagcgaaagaacgattcggtctcctccacagatcgaac 130
FA2ZECP01CQVXB 34  ........................a..g.........c....................t. 93
FA2ZECP01B5XZJ 41  ........................a..g.........c....................t. 100
FA2ZECP01ESN55 39  ........................a..g.........c....................t. 98
FA2ZECP02GTQBY 77  ........................a..g.........c....................t. 136
FA2ZECP02HC6PD 77  ........................a..g.........c....................t. 136
FA2ZECP01BJJLF 77  ........................a..g.........c....................t. 136
FA2ZECP01BUV4K 77  ........................a..g.........c....................t. 136
FA2ZECP01CMQXT 77  ........................a..g.........c....................t. 136
FA2ZECP01EIU4N 77  ........................a..g.........c....................t. 136
FA2ZECP02GDVA8 84  ........................a..g.........c....................t. 143
FA2ZECP01EAXAI 85  ........................a..g.........c....................t. 144
FA2ZECP01AKRDF 65  ........................a..g.........c....................t. 124
FA2ZECP01COKZQ 22  ........................a..g....................a.........t. 81
FA2ZECP02IJLAJ 75  ........................a..g....................a.........t. 134
FA2ZECP02HWQ8M 82  ........................a..g....................a.........t. 141
FA2ZECP02HZUET 83  ........................a..g....................a.........t. 142
FA2ZECP02G3IB6 82  ........................a..g....................a.........t. 141
FA2ZECP02IXW1W 82  ........................a..g....................a.........t. 141
FA2ZECP01CS63B 82  ........................a..g....................a.........t. 141
FA2ZECP01EDQ85 79  ........................a..g....................a.........t. 138
FA2ZECP01EL6BX 190 ........................a..g....................a.........t. 131
FA2ZECP01C8D0X 82  ........................a..g....................a.........t. 141
FA2ZECP01BY5UU 82  ........................a..g....................a.........t. 141
FA2ZECP01D0WL1 81  ........................a..g....................a.........t. 140
FA2ZECP02ISQFN 82  ........................a..g....................a........    138
FA2ZECP02ISLO9 74  ......a.................a..g..............................t. 133
FA2ZECP02F2JZH 73  ......a.................a..g..............................t. 132
```
