# Supplementary material for: Polyploid genome of Camelina sativa revealed by isolation of fatty acid synthesis genes
Source: BMC Plant Biol. 2010 Oct 27;10:233. doi: 10.1186/1471-2229-10-233 (PMC3017853; doi:10.1186/1471-2229-10-233)
Supplement: Additional file 8 — Plant species and sources. List of plant species used and their sources. [file 1471-2229-10-233-S8.DOCX]

**Additional File 8 – Plant species and sources**

| **Species** | **Source** | **Catalogue number** |
| --- | --- | --- |
| *Camelina sativa* Cs32 | USDA | PI 311732 |
| *Camelina sativa* Cs11 | Ames | 26668 |
| *Arabidopsis thaliana,* ecotype Col-0 | ABRC | CS28166 |
| *Arabidopsis lyrata* | ABRC | CS22696 |
| *Camelina laxa* | USDA | PI 650132 |
| *Camelina microcarpa* | wild collection; Harvard Herbarium | number “01-22” |
| *Camelina microcarpa* | USDA | PI 633188 |
| *Capsella bursa-pastoris* | Wild collection; Harvard Herbarium collection | number “08-188” |
| *Capsella rubella* | ABRC | CS22561 |
| *Camelina hispida* var *grandiflora* | Ames | 21324 |
| *Camelina alyssum* | Ames | 26658 |
| *Camelina rumelica* | Ames | 21327 |
